# Deciphering Local Microstrain-Induced Optimization of Asymmetric Fe Single Atomic Sites for Efficient Oxygen Reduction

**DOI:** 10.1007/s40820-025-01783-4

**Published:** 2025-05-26

**Authors:** Peng Zhang, Siying Huang, Kuo Chen, Xiaoqi Liu, Yachao Xu, Yongming Chai, Yunqi Liu, Yuan Pan

**Affiliations:** 1 State Key Laboratory of Heavy Oil Processing, China University of Petroleum (East China), Qingdao, 266580 People’s Republic of China; 2 School of Materials Science and Engineering, Peking University, Beijing, 100871 People’s Republic of China

**Keywords:** Local microstrain, Asymmetric sites, Dynamic mechanism, Single-atom catalysts, Oxygen reduction

## Abstract

Disrupting the symmetric electron distribution of porphyrin-like Fe single-atom catalysts has been considered as an effective way to harvest high intrinsic activity. Understanding the catalytic performance governed by geometric microstrains is highly desirable for further optimization of such efficient sites. Here, we decipher the crucial role of local microstrain in boosting intrinsic activity and durability of asymmetric Fe single-atom catalysts (Fe–N_3_S_1_) by replacing one N atom with S atom. The high-curvature hollow carbon nanosphere substrate introduces 1.3% local compressive strain to Fe–N bonds and 1.5% tensile strain to Fe–S bonds, downshifting the *d*-band center and accelerating the kinetics of *OH reduction. Consequently, highly curved Fe–N_3_S_1_ sites anchored on hollow carbon nanosphere (FeNS-HNS-20) exhibit negligible current loss, a high half-wave potential of 0.922 V vs. RHE and turnover frequency of 6.2 e^−1^ s^−1^ site^−1^, which are 53 mV more positive and 1.7 times that of flat Fe–N–S counterpart, respectively. More importantly, multiple operando spectroscopies monitored the dynamic optimization of strained Fe–N_3_S_1_ sites into Fe–N_3_ sites, further mitigating the overadsorption of *OH intermediates. This work not only sheds new light on local microstrain-induced catalytic enhancement, but also provides a plausible direction for optimizing efficient asymmetric sites via geometric configurations.

**Supplementary Information:**

The online version contains supplementary material available at 10.1007/s40820-025-01783-4.

## Highlights

- Crucial role of local microstrain was deciphered to boost oxygen electrocatalysis via quantitatively riveting asymmetric Fe–N_3_S_1_ sites on carbon hollow nanospheres with specific curvature.
- The local microstrain accelerates kinetics of *OH reduction on Fe–N_3_S_1_, enabling much enhanced intrinsic activity, selectivity and stability toward oxygen electrocatalysis.
- The strained Fe–N_3_S_1_ sites were monitored to transformed into Fe–N_3_–S_1_ sites, further dynamically mitigating the overadsorption of *OH intermediates.

**Supplementary Information:**

The online version contains supplementary material available at 10.1007/s40820-025-01783-4.

## Graphical abstract

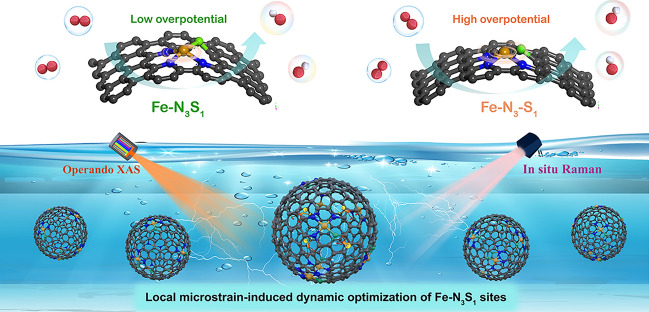

## Introduction

Oxygen reduction reaction (ORR) plays a pivotal role in next-generation energy conversion and storage configurations, such as metal–air batteries and fuel cells. Currently, Pt-group metals (PGM) act as the most efficient electrocatalysts for sluggish cathodic ORR due to their moderate activity [1–3]. However, the modest catalytic durability, terrestrial scarcity and high cost of PGM have hampered the development of such advanced devices. Among various PGM-free electrocatalysts, single-atom catalysts (SACs) have emerged as promising alternatives to PGM in the field of ORR [4, 5]. Accordingly, SACs have become the hottest frontier in energy conversion systems in recent decades [6–8]. Their unique electronic and geometric structures enable maximum atom utilization, optimized adsorption behaviors and tunable catalytic performance. In particular, porphyrin-like Fe–N–C moieties have attracted significant research interest in oxygen-related catalysis due to their prominent activity [9, 10]. Nevertheless, the strong adsorption strength of oxygenated intermediates on symmetric Fe–N_4_ sites leads to a blocked ORR process and increased reaction barriers [11, 12]. Numerous efforts have been developed to address this dilemma by exploiting efficient Fe SACs with asymmetric coordination structure.

The introduction of the secondary atoms into the first coordination shell of Fe–N–C structure could disrupt symmetry of center atoms and achieve satisfactory oxygen activations. Intensive research has been carried out to incorporate heteroatoms (P, S, B, etc.) into the carbon matrix of Fe SACs [13, 14]. Thereinto, the introduction of S atoms to form Fe–N_3_S_1_ sites is prone to result in an improvement of intrinsic ORR activity due to the fact that the asymmetric coordination structure could alleviate overadsorption of oxygenated intermediates on active sites [15, 16]. Despite tremendous efforts, the general try-and-error modes for constructing SACs lack the efficiency to further improve the activity of single-atom catalysts. Most of previous work focused on the fundamental coordinate structure, and the understanding of catalytic behavior relies heavily on flat graphene-based models and experiments instead of real geometric configurations [17, 18]. Compared with flat geometry, high-curvature surface tends to create no-planar curved structure and introduce unique microstrain effect, which might be conducive to boosting catalytic activity [19]. Further optimization of such efficient single atomic sites depends strongly on a comprehensive understanding of the structure–performance relationship and catalytic behavior, especially the dynamic mechanism. This understanding, however, still remains unclear due in part to a lack of quantitative and in-depth exploitation [20, 21].

Herein, we unveil the local microstrain-induced optimization for asymmetric Fe–N_3_S_1_ sites to boost oxygen reduction performance by quantitatively riveting isolated Fe–N_3_S_1_ sites on carbon matrix with specific curvatures. The high-curvature hollow carbon nanosphere was justified to introduce compressive strain to Fe–N bonds and tensile strain to Fe–S bonds. Consequently, Fe–N_3_S_1_ anchored on hollow carbon nanosphere (FeNS-HNS-20) with optimized strained structure exhibited remarkable activity with *E*_1/2_ of 0.922 V vs. RHE and high intrinsic site activity of 6.2 e^−1^ s^−1^ site^−1^, which were 53 mV more positive and 1.7 times that of flat Fe–N–S counterparts, respectively. In addition, rechargeable Zn–air batteries assembled with optimal FeNS-HNS-20 showed a high peak power density of 214 mW cm^−2^ and ultra-long durability of 2200 cycles (1100 h). Based on density function theory (DFT) calculations, the downward shift of the *d*-band center of Fe atoms regulated by local microstrain could accelerate kinetics of *OH reduction and reduce the energy barrier for ORR. More importantly, operando X-ray absorption spectroscopy and in situ Raman results monitored that the highly curved Fe–N_3_S_1_ sites undergo a dynamic structural evolution to Fe–N_3_ by breaking stretched Fe–S bond, thereby mitigating the overadsorption of *OH. The stable compressed Fe–N bonds were therefore retained, which guaranteed the high durability. This work provides a thorough understanding of structure–activity relationship of asymmetric Fe sites and highlights the significance of geometric configurations, which paves the way for the design of efficient SACs.

## Experimental Section

### Preparation of Materials

#### Preparations of FeNS-NS

In a typical synthesis, 1 g of coal tar pitch and 1 g of melamine were dispersed in 20 mL of N,N-dimethylformamide (DMF) and followed by sonication for 30 min to form solution A. Subsequently, 50 mg of hemin chloride was dispersed into 40 mL of DMF and sonicated for 30 min to form solution B. Then solution B was dropped into solution A and stirred for 24 h. The mixed solution was evaporated at 120 °C to remove the solvent. The resulted precursors were heated to 900 °C at NH_3_ atmosphere for 2 h with a heating rate of 5 °C min^−1^. The prepared catalysts were washed with 0.5 M H_2_SO_4_ to remove possible metal particles and dried in a vacuum oven at 60 °C to obtain the final catalysts FeNS-NS.

#### Preparations of FeNS-HNS-X

The synthesis of FeNS-HNS was similar to FeNS-NS except that 1 g of SiO_2_ with a diameter of 20 nm was added into solution A. The resultant pre-catalysts after pyrolysis were collected and followed by 5% HF washing for 12 h. Then the catalysts were washed with deionized water to remove excessive HF and dried in a vacuum oven at 60 °C to obtain the final catalysts FeNS-HNS-20.

To prepare FeNS-HNS-180, SiO_2_ nanoparticles with a diameter of around 176 nm were synthesized based on modified Stöber method [22]. In a typical synthesis, 15 mL of ethanol, 5 mL of deionized water and 0.7 mL ammonia aqueous solutions (28%) were mixed and stirred for 30 min. Subsequently, 0.6 mL of TEOS was quickly injected into the mixed solutions and stirred for 1 min at 1100 r min^−1^. The above solutions were stirred at 400 r min^−1^ for 10 h. The obtained products were then centrifuged and heated in a vacuum at 60 °C. The preparation process of FeNS-HNS-180 was analogous to that of FeNS-HNS-20 except that 20 nm of SiO_2_ was replaced by 176 nm of SiO_2_.

The details of structural characterization, electrochemical measurements and theoretical calculations are provided in the supplementary information.

## Results and Discussion

### Characterization of Strained Asymmetric Fe SACs

**Fig. 1:**
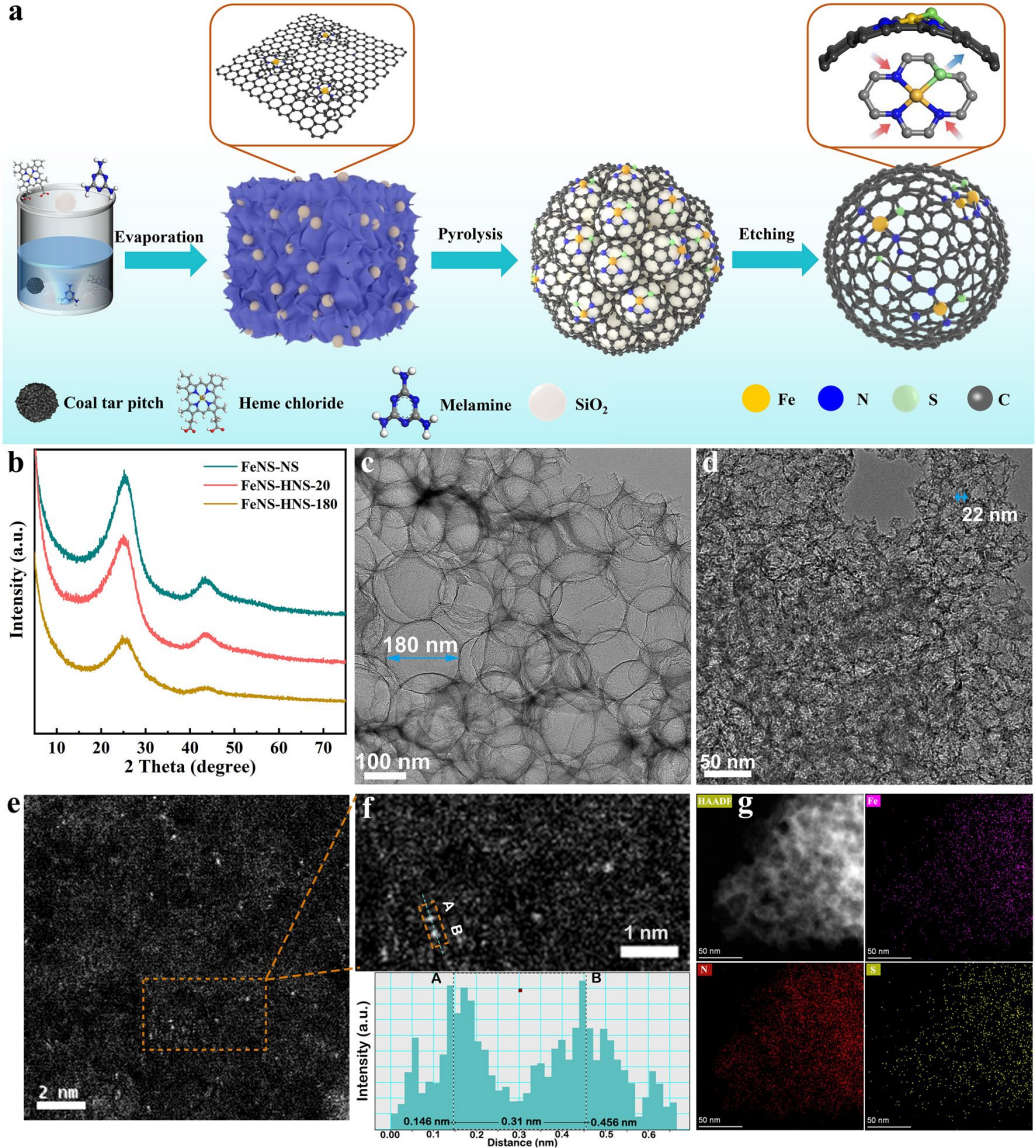
Synthesis and structure characterization of FeNS-HNS. **a** Schematic illustration of synthesis process. **b** XRD. **c, d** TEM for FeNS-HNS-180 and FeNS-HNS-20, respectively. **e** and **f** Aberration-corrected (AC)-HAADF-STEM and magnified AC-HAADF-STEM image for FeNS-HNS-20, respectively. **g** HAADF-STEM and element mapping of Fe, N, S for FeNS-HNS-20

The synthesis of Fe SACs on hollow carbon nanospheres is schematically illustrated in Fig. 1a. Here, coal tar pitch (CTP), consisting of polycyclic aromatic hydrocarbons (PAHs), was selected as carbon and sulfur source due to its high conductivity and excellent flexibility, which enabled encapsulating templates to form a highly curved surface [23, 24]. Moreover, the π-π stacking between PAHs and Fe precursors of heme chloride macrocycle also contributed to the dispersion of Fe atoms [25]. Herein, Fe moieties immobilized on hollow carbon nanosphere (FeNS-HNS-x) with different curvatures were constructed after removing SiO_2_ templates, where x represents the diameter of nanospheres. For comparison, Fe atoms anchored on carbon nanosheets (FeNS-NS) were also prepared without SiO_2_ as templates. The detailed synthetic procedure is provided in the supporting information.

X-ray diffraction (XRD) technique was conducted to identify phase information of as-prepared catalysts. As depicted in Fig. 1b, all the catalysts exhibited two broad peaks at around 24° and 44°, corresponding to (002) and (101) crystal face of graphitic carbon, respectively. No characteristic peaks related to Fe species (e.g., metallic Fe, Fe_3_C, FeS_x_, FeO_x_) can be observed, implying the high dispersion of Fe atoms. The microstructure and morphology of catalysts were disclosed by transmission electron microscopy (TEM) and atomic force microscope (AFM). The morphology of the transparent center with thick edges confirmed the hollow nature of FeNS-HNS as observed in Fig. 1c, d. Diameters of FeNS-HNS-180 and FeNS-HNS-20 were estimated to be around 180 and 22 nm (Figs. S1 and S2), respectively. The dependence of hollow carbon nanospheres size on templates demonstrates the advance of CTP precursors for catalysts geometry and strain control (Fig. S3). The unique curved surface would regulate the electronic structure of isolated Fe SACs owing to geometric bendings [26, 27]. No aggregation of Fe-containing nanoparticles was observed at the randomly selected TEM, demonstrating the highly dispersed Fe atoms. Unlike the scenarios of FeNS-HNS, FeNS-NS without SiO_2_ as templates exhibited a 2-dimensional sheet-like morphology with a thickness of 3.02 nm as shown in AFM and TEM image of Figs. S4 and S5. Raman spectra were then conducted to characterize the degree of defects for as-prepared catalysts. The characteristic peak at 1343 cm^−1^ denotes the disordered D-band signal, while the peak at 1605 cm^−1^ corresponds to the ordered graphitic carbon G band. Therefore, the D-G band (*I*_D_/*I*_G_) ratios were applied as an indicator to clarify the degree of defects. The analogous intensity of *I*_D_/*I*_G_ for as-prepared catalysts as exhibited in Fig. S6 indicated the similar degree of defects, due to the same CTP precursor and preparation process. The approximate degree of defects would rule out their contribution to intrinsic ORR activity for Fe sites.

The curvature of different geometry can be quantified via *K* = 1/*R*, where *R* is the radius of the structure models. The curvatures of FeNS-HNS-20 (R = 11 nm), FeNS-HNS-180 (*R* = 90 nm) and FeNS-NS are calculated to be 0.091, 0.011 and 0, respectively. Compared to flat graphene-like carbon nanosheets, the hollow carbon nanospheres possessed more strained C–C/C–N bonds due to geometric bending [28, 29]. The strained substrates possibly give rise to distinctive electronic regulation and dynamic evolution to active centers, thus manipulating catalytic process of SACs [26, 30]. Spherical aberration-corrected high-angle annular dark-field scanning TEM (AC-HAADF-STEM) with subangstrom resolution was further equipped to identify the dispersion of Fe atoms. The isolated Fe atoms presented as bright dots were permeated in the carbon matrix as exhibited in Fig. 1e. The distance between adjacent Fe atoms in the selected area was determined to be 0.31 nm, as exhibited in the enlarged AC-HAADF-STEM images in Fig. 1f. The uniformly dispersed Fe atoms as well as the presence of N and S were also evidenced by the HAADF-STEM and corresponding elemental mapping of Figs. 1g, S7 and S8, suggesting the efficient merits of CTP substrates in dispersing Fe atoms.

**Fig. 2:**
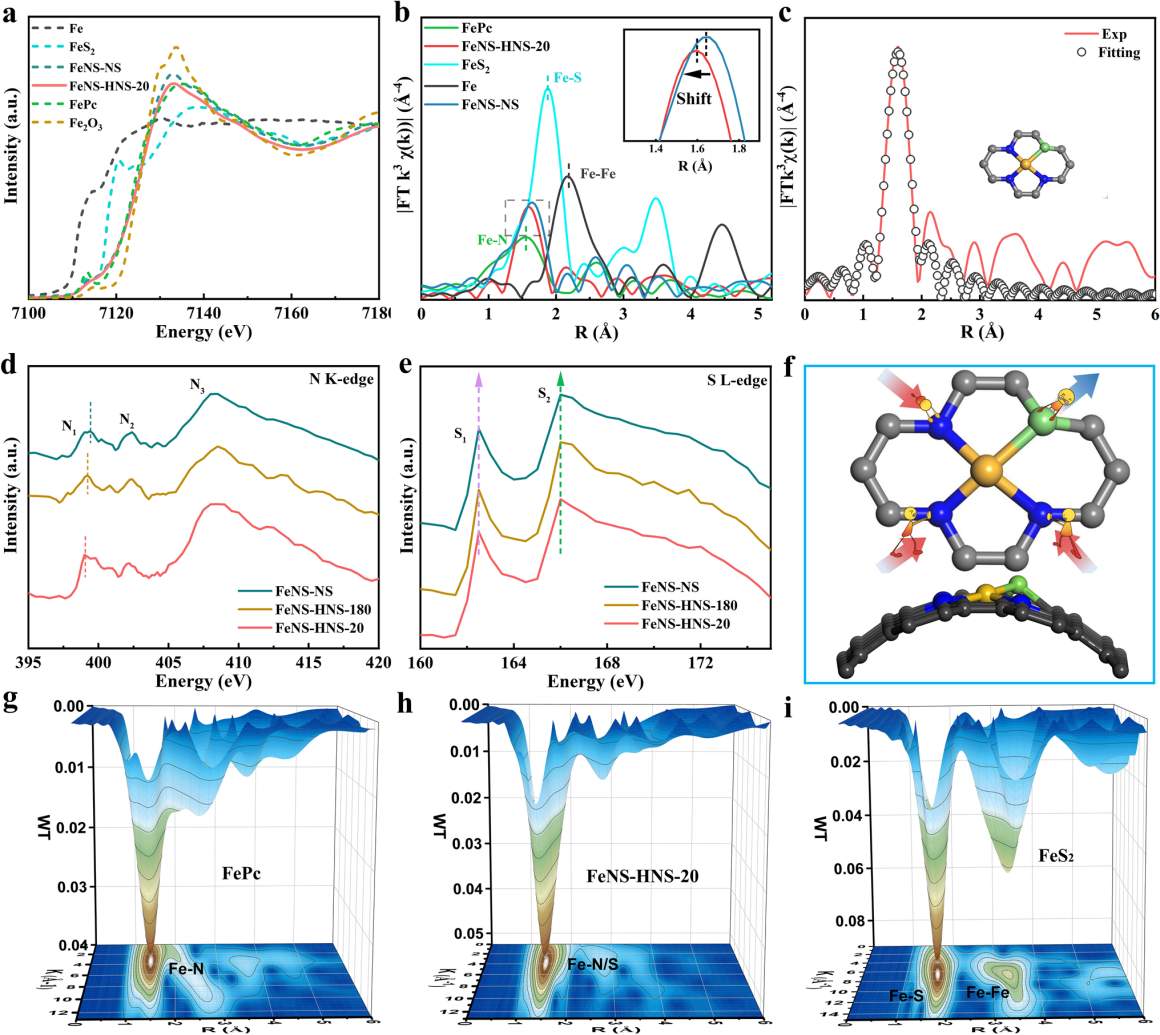
Electronic structure and local chemical configurations of catalysts. **a** Fe K-edge XANES spectra of FeNS-HNS-20, FeNS-NS and references. **b** FT-EXAFS k^3^-weighted Fe K-edge of FeNS-HNS-20 and references. **c** EXAFS fitting curves of FeNS-HNS-20 in R space. **d** N K-edge and **e** S L-edge of as-prepared catalysts. **f** Schematic illustration of multilevel local strain on curved Fe–N_3_S_1_ sites. The carbon, nitrogen, iron and sulfur atoms are marked black, blue, yellow and green, respectively. **g**–**i** Wavelet transform of Fe K-edge EXAFS of FeNS-HNS-20 and references

X-ray photoelectron spectroscopy (XPS) was then performed to evaluate the surface elemental composition of as-prepared catalysts. The bonding status of N and S for as-prepared catalysts can be analyzed by high-resolution N 1*s* and S 2*p* spectra. As revealed in Fig. S9, the N 1*s* spectra for as-prepared catalysts can be deconvoluted into peaks at around 397.9, 399.3, 400.5, 401.2 and 404.1 eV, which can be assigned to pyridinic N, Fe–N, pyrrolic N, graphitic N and oxidized N, respectively[25]. The S 2*p* spectra of as-prepared catalysts in Fig. S10 can be divided into peaks for C-S-C (164 eV for S 2*p*_3/2_, 165.2 eV for S 2*p*_1/2_), C-SO_x_-C (168.5 eV for S 2*p*_3/2_, 169.7 eV for S 2*p*_1/2_) [13, 18]. Notably, the characteristic peak at 161.5 eV can also be observed, indicating the form of Fe–S bond [15]. The XPS results corroborate that the N and S heteroatoms have been successfully doped into carbon matrix, forming stable bonds with Fe atoms. X-ray absorption spectroscopy (XAS) was further performed to detect the electronic structure and local configuration of catalysts. As illustrated in Fe K-edge X-ray absorption near-edge structure (XANES) spectra in Fig. 2a, the absorption edge of FeNS-HNS-20 is observed to be much closer to the edge of FePc, suggesting an Fe oxidation state of + 2 in FeNS-HNS-20. Note that the absorption edge of highly curved FeNS-HNS-20 was observed to slightly incline to higher energy compared to flat FeNS-NS, indicating an increased oxidation state and the crucial role of local substrate strain in regulating electronic structure. A pre-edge peak of Fe K-edge XANES for FePc located at around 7114 eV can also be identified, originating from the square planar *D*_*4h*_ of Fe-centered coordination due to 1*s* to 4*p*_*z*_ transition, which is considered to be the fingerprint of porphyrin-like planar Fe–N_4_ [31]. Compared to FePc with typical planar Fe–N_4_ coordination, FeNS-HNS-20 and FeNS-NS exhibited attenuated intensity of pre-edge peak, implying a distorted *D*_*4h*_ symmetry. The distorted coordination structure might be ascribed to the geometric distortion by forming Fe–S bond due to the rich S source of CTP [24]. Fourier transformed k^3^-weighted Fe K-edge extended X-ray adsorption fine structure (FT-EXAFS) spectra of FeNS-HNS-20 showed an enlarged peak at 1.60 Å (Fig. 2b). The peak locates between the Fe–N scattering path of FePc (1.53 Å) and standard Fe–S scattering path of FeS_2_ (1.87 Å), suggesting a hybrid coordinate structure of Fe–N and Fe–S bonds [32]. Further fitting results of Fe moiety in both R and K space as shown in Figs. 2c and S11 disclosed a Fe–N_3_S_1_ asymmetric coordinate structure with Fe–N coordination number (CN) of 2.9 and Fe–S CN of 0.9 (Table S1) for FeNS-HNS-20, which was also consistent with XPS results. It was noteworthy that a negative shift in the scattering path from 1.63 Å for flat FeNS-NS to 1.60 Å for high-curvature FeNS-HNS-20 can be observed in the enlarged view of Fig. 2b, indicating integrally compressed Fe–N/S bonds.

Simulated calculations were then carried out to decipher the effect of substrate strain on the asymmetric coordinate structure. As shown in Figs. S12–S14, the optimized structure disclosed an integrally compressed tendency for coordinate bonds with the increasing of curvature from 0 to 0.091, which was in good agreement with the XAS results [19]. In detail, a compressive tendency for Fe–N bonds and a tensile tendency for Fe–S bonds of asymmetric Fe–N_3_S_1_ sites with the increase of substrate curvature can be witnessed as summarized in Fig. S15. Compared to flat FeNS-NS, the Fe–N bonds in FeNS-HNS-20 are compressed by 1.3%, while the Fe–S bond is elongated by 1.5%, suggesting multilevel local microstrain is created on asymmetric Fe–N_3_S_1_ sites by curved structure. In addition, no scattering path of Fe–Fe peak at 2.2 Å can be observed, demonstrating the isolated Fe atoms in carbon matrix. Wavelet transform extended X-ray absorption fine structure (WT-EXAFS) analysis was allowed to differentiate lighter and heavier backscattering atoms even they are nearly equidistant from the central atoms. By comprehensive consideration of Fe–N and Fe–S contributions, the WT contour plots of FeNS-HNS-20 exhibited a maximum peak at 3.9 Å^−1^, lying between those of FePc and FeS_2_ as shown in Fig. 2g-i. The higher wavenumber of FeNS-HNS-20 compared to FePc (3.4 Å^−1^) indicates the presence of heavier backscattering atoms, such as S, than N atoms in the Fe moieties.

Synchrotron radiation-based soft XANES of nitrogen K-edge and sulfur L-edge was further performed to probe the electronic and atomic interplay between coordinate atoms and center Fe atom in Fe–N–S catalysts. As shown in Fig. 2d, three distinct peaks in N K-edge XANES appeared in the region of 397 to 410 eV, which can be assigned to the pyridinic N/Fe–N, graphitic N and σ* resonance, respectively [32]. The rich pyridinic N was conducive to anchoring Fe atoms through strong interactions between empty *d*-orbitals of metal ions and lone electron pairs of N atoms [33]. A negative shift of N_1_ and N_2_ peaks can also be observed with the increase of curvature-induced strain, which results from more electron transfer between Fe and coordinate N atoms in high-curvature hollow carbon nanosphere than in carbon nanosheets [34]. The regulated electronic structure of central Fe atoms is closely related to the adsorption behavior of oxygenated intermediates during ORR processes. With regard to S L-edge XANES, two obvious peaks of S_1_ and S_2_ can be observed in Fig. 2e. The former one can be assigned to C-S-C coordination species in carbon skeleton, while the later one is attributed to the Fe–S bond, in good accordance with Fe XANES analysis [35–37]. Interestingly, no obvious shift of S L-edges of all the as-prepared catalysts can be identified in Fig. 2e, which could be attributed to the lattice distortion after doping of larger S atoms in carbon matrix and is well consistent with the optimized structure in the above simulations [38, 39]. The lattice distortion would drive S atoms out of Fe–N plane**,** leading to implicit changes with the increase of curvature-induced strain. The local microstrain engineering and optimized geometric configuration of curved asymmetric Fe–N_3_S_1_ sites is schematically illustrated in Fig. 2f. The XANES and theoretical simulation results together demonstrate that isolated Fe–N_3_S_1_ sites with curved structure and local microstrain are successfully achieved in FeNS-HNS-20. The local microstrain induced by geometric configuration of asymmetric sites might give rise to further dynamic optimization compared with monotonous Fe–N coordinate structure [15].

### Electrochemical Performance Evaluation

**Fig. 3:**
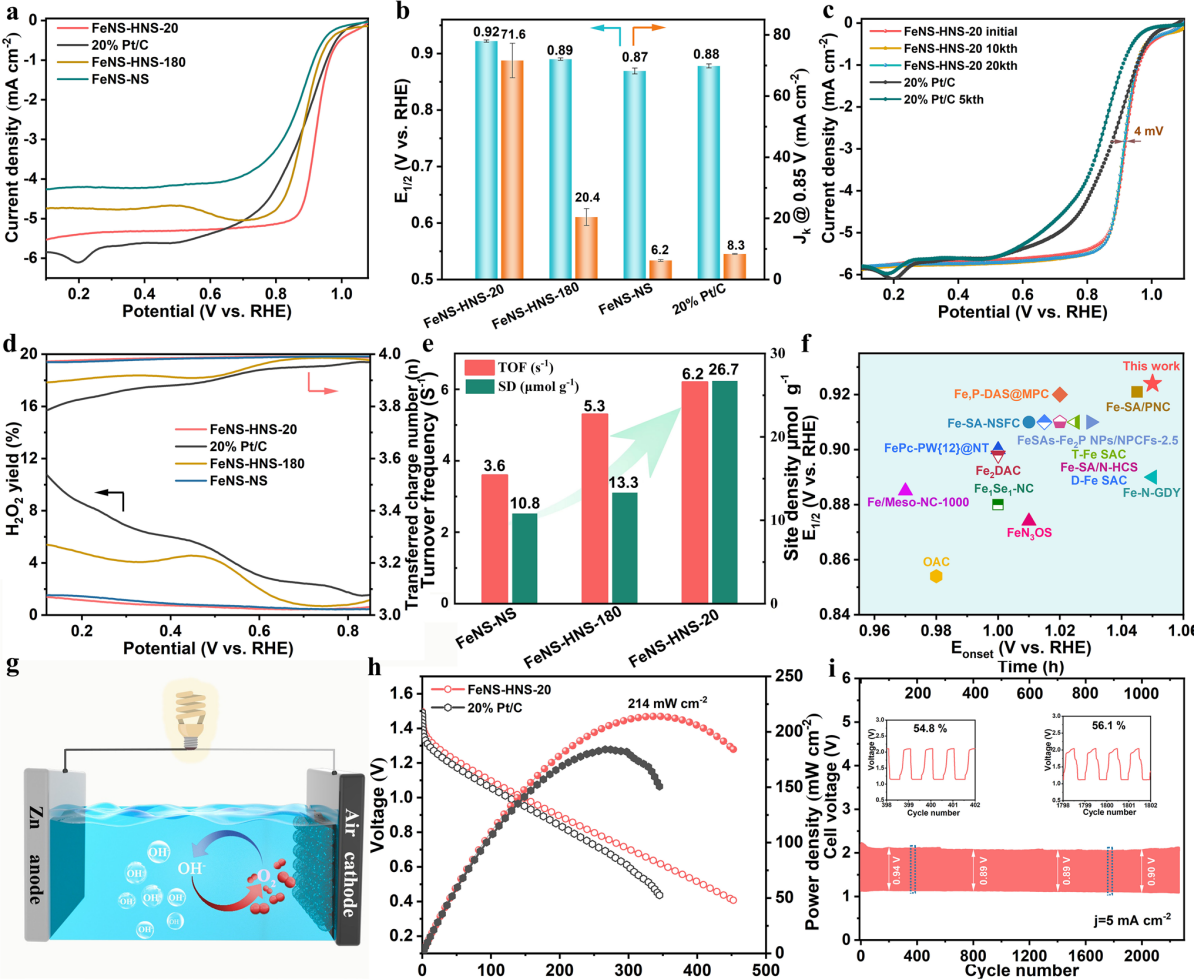
Electrocatalytic performance of catalysts. **a** LSV curves tested in O_2_-saturated 0.1 M KOH. **b** Half-wave potential and kinetic current density at 0.85 V. **c** Accelerated durability tests of FeNS-HNS-20 and 20% Pt/C, respectively. **d** H_2_O_2_ yield and transferred charge number. **e** Turnover frequency and site density. **f** ORR activity of FeNS-HNS-20 compared with references. **g** Schematic illustration for rechargeable zinc–air batteries. **h** Polarization curves and corresponding power density of ZABs. **i** Galvanostatic cycling curves at a current density of 5 mA cm^−2^ of ZABs with FeNS-HNS-20 electrocatalysts

The ORR performance of electrocatalysts was scrutinized using a rotating disk electrode (RDE) on a typical three-electrode system in 0.1 M KOH medium. The ORR performance for as-prepared catalysts was determined based on three independent experiments (Figs. S16–S19). All the voltages have been converted to reversible hydrogen electrode (RHE) voltages unless otherwise stated. As shown in Fig. 3a, FeNS-HNS-20 exhibited the most positive LSV curves compared to 20% Pt/C benchmarks and Fe–N–S counterparts. Specifically, FeNS-HNS-20 achieved a higher half-wave potential (*E*_1/2_) of 0.922 V and onset potential (*E*_onset_) of 1.05 V, which outperformed Pt/C (*E*_1/2_ of 0.878 V, *E*_onset_ of 1.03 V), FeNS-HNS-180 (*E*_1/2_ of 0.890 V, *E*_onset_ of 1.01 V) and FeNS-NS (*E*_1/2_ of 0.869 V, *E*_onset_ of 1.00 V). Impressively, FeNS-HNS-20 with a highly curved structure was 32 and 53 mV more positive of *E*_1/2_ than FeNS-HNS-180 and FeNS-NS, respectively, demonstrating the high efficiency of local microstrain engineering. Moreover, the merits of local microstrain in regulating catalytic behavior for oxygenated electrocatalysis on asymmetric Fe–N_3_S_1_ sites were also witnessed by oxygen evolution reaction (OER). As shown in Fig. S20, FeNS-HNS-20 achieved a potential of 1.584 V to reach a current density of 10 mA cm^−2^, which outperformed those of FeNS-HNS-180 (1.650 V) and FeNS-NS (1.704 V). The kinetic current density (*J*_k_) at 0.85 V was also performed to evaluate the kinetic activity of electrocatalysts. As shown in Fig. 3b, FeNS-HNS-20 showed the highest *J*_k_ of 71.6 mA cm^−2^ based on Koutecky–Levich (K-L) equation calculation due to a favorable ORR process, which was 3.5 and 11.5 times those of FeNS-HNS-180 and flat FeNS-NS, respectively. The high value of kinetic current density guarantees a fast ORR process for FeNS-HNS-20, demonstrating the advantage of local microstrain engineering in regulating catalytic behavior. Meanwhile, FeNS-HNS-20 also outperformed the counterparts (2.1 times of FeNS-HNS-180 and 2.6 times of FeNS-NS) according to the normalized kinetic activity by amount of Fe atoms and specific area determined by inductively coupled plasma optical emission spectroscopy (ICP-OES, Table S2) and specific surface area, respectively, as exhibited in Figs. S21 and S22. The normalized kinetics demonstrated a structure-dependent catalytic kinetics of asymmetric Fe–N_3_S_1_ sites stemming from local geometric strain.

The durability of electrocatalysts was also a crucial parameter with regard to the potential for practical applications [40, 41]. FeNS-HNS-20 showed a high durability with negligible *E*_1/2_ loss (4 mV) after 20 k CV cycles during accelerated durability test (ADT) as shown in Fig. 3c, which could be ascribed to the stable shortened Fe–N bonds induced by local microstrain [42, 43]. Different from scenario of FeNS-HNS-20, the benchmark 20% Pt/C exhibited significant attenuation with 40 mV loss of *E*_1/2_ only after 5 k CV cycles during ADT. And the excellent durability of FeNS-HNS-20 was also visualized by long-term i-t chronoamperometric tests as exhibited in Fig. S23. The negligible changes for Fe and N states (Figs. S24–S26) before and after stability tests further verify the stable of asymmetric Fe–N_3_S_1_ sites in FeNS-HNS-20. FeNS-HNS-20 achieved a current retention of 97.7% after 50,000 s test, which surpassed that of commercial 20% Pt/C of 73.6%. Apart from the long-term stability, the methanol crossover effect was also evaluated by instantaneously injecting methanol into O_2_-saturated 0.1 M KOH solution during i-t chronoamperometric tests. FeNS-HNS-20 exhibited no disturbance of current after injection of methanol at 400 s as shown in Fig. S27, while Pt/C underwent a sharp current loss, showing the prominent methanol tolerance ability of highly curved asymmetric Fe–N_3_S_1_ active sites. Moreover, a favorable 4-electron ORR pathway of FeNS-HNS-20 was also evidenced by its low H_2_O_2_ yield of < 2% over a wide potential range of 0.1–0.9 V recording on a rotating ring disk electrode (RRDE), corresponding to a high electron transfer number of 3.96–3.97 in Fig. 3d. The much more selective ORR process of FeNS-HNS-20, compared to FeNS-HNS-180, confirmed the effectiveness of the local microstrain engineering in regulating ORR process. The superior alkaline ORR activity of FeNS-HNS-20 was witnessed in comparison with recently reported Fe-based SACs in *E*_1/2_-*E*_onset_ map as depicted in Fig. 3f and Table S3. These results demonstrated that FeNS-HNS-20 can efficiently catalyze four-electron ORR under alkaline conditions.

Conventional RDE test was severely restricted by the low oxygen solubility in electrolyte and geometric structure of catalysts, resulting in a mass transport-controlled apparent performance, while the in situ electrochemical method by means of nitrite absorption followed by reductive stripping enables the reactants transport similar to the active sites [44]. The average intrinsic activity of single site (turnover frequency, TOF) and site density (SD) can be quantitatively evaluated based on stripping charge regardless the geometry of the catalysts as shown in Figs. S28–S30 [45, 46]. As a result, FeNS-HNS-20 demonstrated the highest site activity with TOF of 6.2 e^−1^ s^−1^ site^−1^ at 0.80 V (Fig. 3e), which outperformed most recently reported Fe SACs [25, 47, 48]. In addition, the TOF of FeNS-HNS-20 was 1.2 and 1.7 times those of FeNS-HNS-180 (5.3 e^−1^ s^−1^ site^−1^) and flat FeNS-NS (3.6 e^−1^ s^−1^ site^−1^), respectively. The intrinsic ORR activity of Fe–N_3_S_1_ sites discloses a positive response with the increase of substrate strain, indicating a substrate strain-governed ORR activity on specific Fe–N_3_S_1_ asymmetric sites. The crucial role of local microstrain engineering in regulating catalytic performance of active Fe–N_3_S_1_ sites might explicate the reason for ORR discrepancy of the active sites with same coordinate structure. Besides, the highly curved nanosphere structure of FeNS-HNS-20 also contributed to enhance the apparent ORR activity due to the high site density. The above results strongly suggested a higher average intrinsic activity of highly strained Fe–N_3_S_1_ for ORR of FeNS-HNS compared to that in flat FeNS-NS, which highlights the significance of local microstrain engineering and geometric configurations.

The prominent performance of oxygen electrocatalysis for FeNS-HNS-20 promises its practical application in oxygen/air-related energy conversion devices [49]. Stacked-type zinc–air battery (ZAB) with FeNS-HNS-20 as air cathodes was assembled as illustrated in Fig. 3g. As shown in Fig. 3h, ZABs assembled with FeNS-HNS-20 delivered a peak power density of 214 mW cm^−2^, outperforming the ZABs armed with benchmark Pt/C electrocatalysts (184 mW cm^−2^). More importantly, rechargeable ZABs with FeNS-HNS-20 as air cathodes also show a high open-circuit voltage (OCV) of 1.552 V (Fig. S31) and superb cycling stability with no obvious voltage loss in 2200 cycles (1100 h, Fig. 3i). The stable charging/discharging voltage efficiencies were calculated to be 54.8–56.1% during the whole lifespan. Moreover, the ZAB assemble with FeNS-HNS-20 also remained negligible decay under a higher current density of 10 mA cm^−2^ in 500 cycles of discharging/charging tests (Fig. S32), suggesting a reliable prospect.

### Theoretical Evidence of Microstrain-induced Catalytic Behavior

**Fig. 4:**
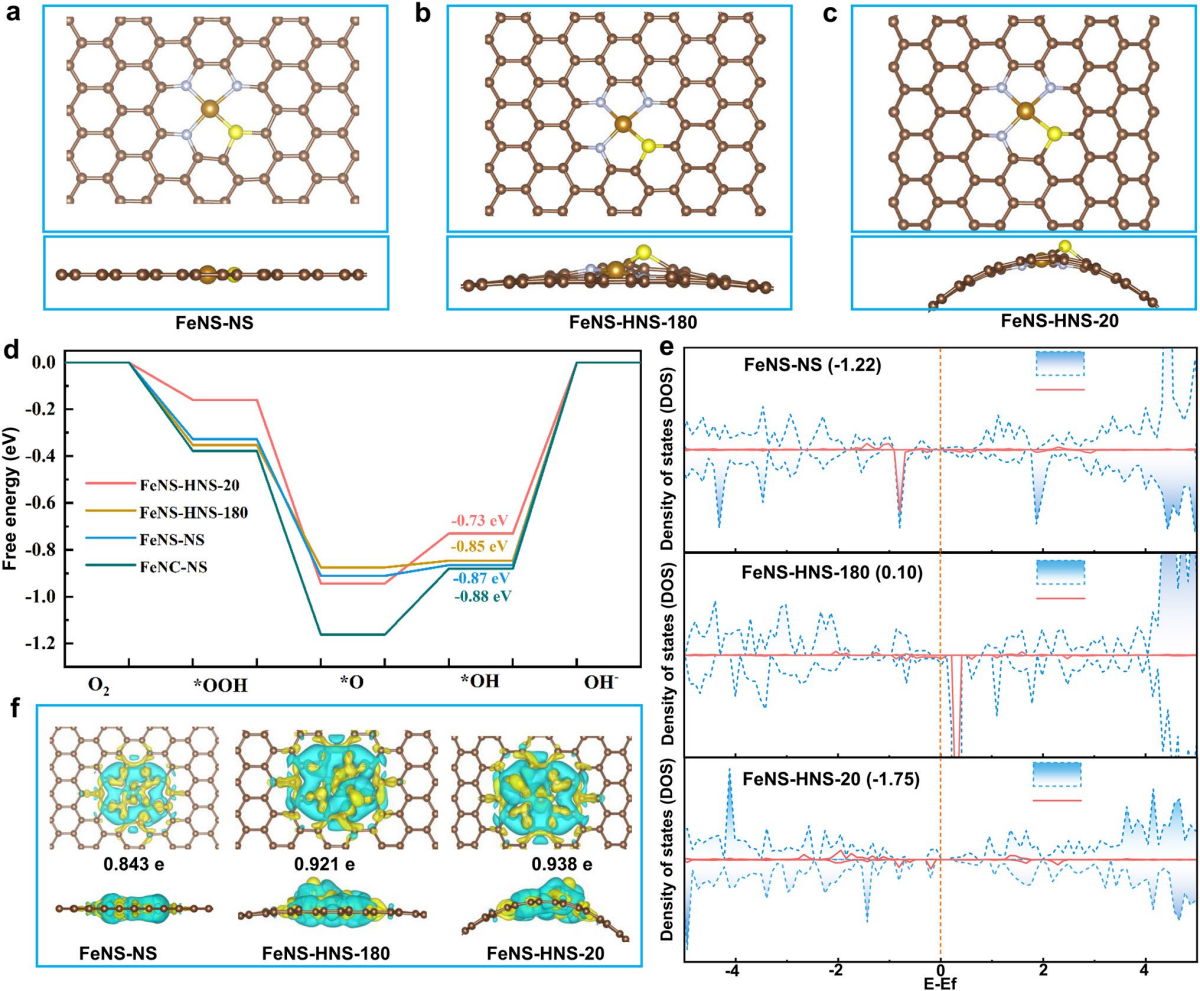
DFT calculations. **a**–**c** The optimized geometric structure for FeNS-NS, FeNS-HNS-180 and FeNS-HNS-20, respectively. **d** Free energy profiles for ORR on FeNC-NS, FeNS-NS, FeNS-HNS-180 and FeNS-HNS-20 at equilibrium potential of 1.23 V. **e** Total and partial density of state for Fe *d*-orbitals for FeNS-NS, FeNS-HNS-180 and FeNS-HNS-20, respectively. **f** The differential charge density of FeNS-NS, FeNS-HNS-180 and FeNS-HNS-20 (yellow: electron accumulation; cyan: electron depletion)

To elucidate the origin of high intrinsic ORR activity and gain a deeper understanding of the concrete contribution of substrate strain, density functional theory (DFT) calculations were carried out. Several optimized models were constructed as demonstrated in Fig. 4a–c, including Fe–N_3_S_1_ sites confined in carbon substrates with curvatures of 0, 0.011 and 0.091, respectively, based on the real geometry observed above. Particularly, theoretical calculations of conventional Fe–N_4_ sites on flat graphene nanosheets (FeNC-NS, Fig. S33) were also performed for comparison.

The calculations were conducted following a typical four-electron ORR pathway involving different reaction intermediates, such as *OOH, *O and *OH, as shown in Figs. S33–S36 [50, 51]. Compared with pristine Fe–N_4_ model, all asymmetric Fe–N_3_S_1_ configurations demonstrated higher ORR activity as shown in Fig. 4d. The desorption of *OH was determined to be the rate-determining steps (RDS) for active sites. FeNS-HNS-20 with 1.3% compressed Fe–N bonds and 1.5% stretched Fe–S bond exhibited the highest ORR activity with *OH desorption energy of 0.73 eV, followed by FeNS-HNS-180 (0.85 eV) and FeNS-NS (0.87 eV). That is to say, the local microstrain engineering could accelerate kinetics of *OH reduction on asymmetric Fe–N_3_S_1_ sites by lowering reaction barriers. Besides, the free energy changes from *OOH to *O were −0.79, −0.52 and −0.58 eV for FeNS-HNS-20, FeNS-HNS-180 and FeNS-NS, respectively. The rapid transformation of *OOH on highly strained FeNS-HNS-20 guarantees the high 4-e^−^ ORR selectivity, which is consistent with the RRDE tests in Fig. 3d. The total and partial density of states (PDOS) were conducted to further disclose the effect of local microstrain on *d*-band center for asymmetric Fe–N_3_S_1_ sites. As shown in Fig. 4e, the *d*-band centers of Fe atoms for FeNS-HNS-20, FeNS-HNS-180 and FeNS-NS were calculated to be − 1.75, 0.10 and − 1.22 eV, respectively. The downward shift *d*-band centers of Fe atoms in FeNS-HNS-20 induced by local strain were conducive to mitigating excessive adsorption of the oxygenated intermediates on Fe–N_3_S_1_ sites during ORR process, which was consistent with free energy analysis. The differential charge density and Bader charge analysis were then conducted to determine the influence of local microstrain on electronic interactions between central Fe and coordinate atoms as shown in Fig. 4f. The Bader charge analysis of Fe in highly curved FeNS-HNS-20 is larger (0.938 e^−^) in comparison with FeNS-HNS-180 (0.921 e^−^) and FeNS-NS (0.843 e^−^), demonstrating a more obvious charge transfer. The simulated results were also in line with electronic interplays observed in Fig. 2a, d.

Based on above characterization and calculation results, the local microstrain originated from geometric bendings would regulate the catalytic behavior of asymmetric Fe–N_3_S_1_ sites. As a result, the overly strong bonded oxygenated intermediates on Fe sites, which are the main reason that limits the intrinsic activity of Fe SACs, are avoided for curved Fe–N_3_S_1_ sites, thereby generating enhanced activity.

### Investigation of Dynamic Optimization on Strained Fe–N_3_S_1_ Sites

**Fig. 5:**
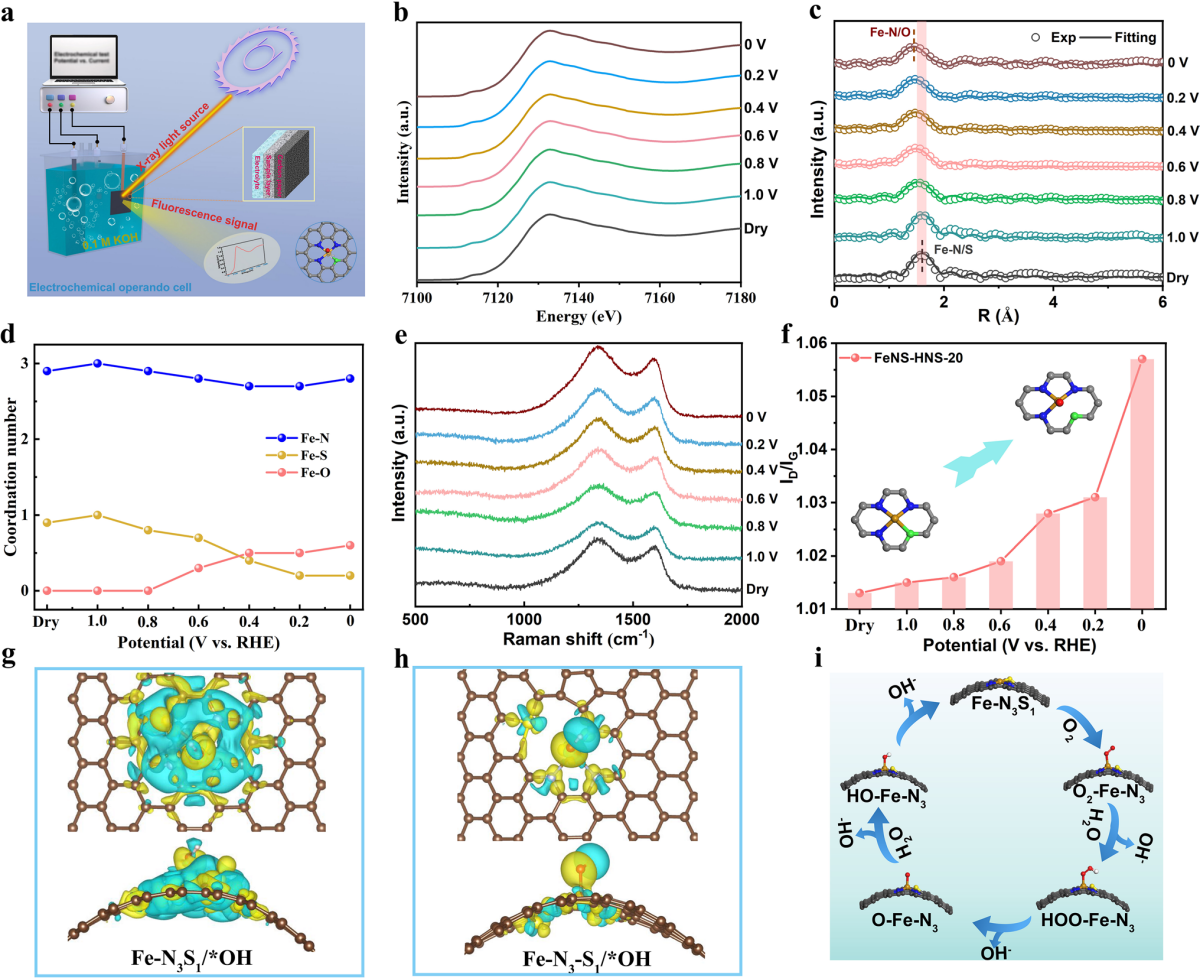
Operando and in situ characterization for FeNS-HNS-20. **a** Schematic illustration for operando XAS. **b** Operando Fe K-edge XANES spectra for FeNS-HNS-20 tested in O_2_-saturated 0.1 M KOH. **c** Experimental (Exp) and fitting (Fitting) curves of FT-EXAFS k^3^-weighted Fe K-edge for FeNS-HNS-20 under working conditions. **d** Fe–N, Fe–S and Fe–O coordination number for FeNS-HNS-20 at different working potentials. **e** In situ Raman spectroscopy of FeNS-HNS-20 at different working potentials. **f** *I*_D_/*I*_G_ values under different working potentials. **g, h** The differential charge density of Fe–N_3_S_1_ sites with adsorption of *OH in FeNS-HNS-20 before and after Fe–S bond breakage, respectively. **i** Dynamic ORR mechanisms on strained Fe–N_3_S_1_ sites under high overpotentials

The dynamic catalytic behaviors provide valuable information on the identification of active sites and comprehensive understanding of reaction insights [52]. Operando XAS was carried out to monitor the dynamic structure evolution of active sites under working conditions as illustrated shown in Fig. 5a, b. The FT-EXAFS analysis in Fig. 5c showed a decreased distance in R space with increasing of overpotentials, suggesting a possible disruption of stretched Fe–S bond in asymmetric Fe–N_3_S_1_ moieties under high overpotentials.

Further fitting results in Figs. 5d, S37 and Table S4 demonstrated the CN of Fe–S bond would transform from initial 0.9 of dry samples to 0.2 under high overpotentials of 0 V, indicating the breakage of Fe–S bond at high overpotentials. This dynamic evolution could be ascribed to the unique Fe–S coordinate structure in strained FeNS-HNS-20 as revealed by XAS, where the Fe–S bond would be elongated due to substrate strain and extend out of the Fe–N planes. The dynamic evolution of highly strained Fe–N_3_S_1_ would further tune the electronic structure of central Fe atoms and therefore regulate adsorption behavior of oxygenated intermediates during ORR. The differential charge density and Bader charge analysis were accordingly performed to disclose the influence of such dynamic evolution on catalytic behavior. As the desorption of *OH was the RDS for ORR on strained Fe–N_3_S_1_ sites, the charge interactions between *OH and Fe–N_3_S_1_ sites before and after breakage of Fe–S bond were calculated as shown in Fig. 5g, h. In the case of *OH adsorption, central Fe atoms in Fe–N_3_S_1_ sites will lose 1.130 e^−^ and the oxygen will gain 1.050 e^−^. The scenario will be different after the disruption of Fe–S bond. After Fe–S bond breakage, the central Fe atoms will lose 1.087 e^−^ and the oxygen will get 0.942 e^−^. The attenuated charge interactions between Fe and O atoms will optimize the ORR catalytic behavior and facilitate the desorption of *OH. Therefore, the Fe–N_3_–S_1_ sites (by breaking Fe–S bonds) were identified as the real active sites for highly strained Fe–N_3_S_1_ (FeNS-HNS-20) under high working overpotentials due to a more favorable ORR process as depicted in Fig. 5i. In addition, as a result of local microstrain engineering, the stable compressed Fe–N bonds were retained under high overpotentials, which ensured an efficient and durable 4-e^−^ ORR process [42, 43].

Given that the solo dynamic evolution results obtained by operando XAS characterization, in situ Raman spectroscopy method was performed to surveil structural transformation of FeNS-HNS-20, as shown in Fig. 5e. The intensity ratios of *I*_D_*/I*_G_ as summarized in Fig. 5f were observed to incline to higher values with the increase of applied overpotentials, suggesting a heavy dislocation of carbon matrix under ORR working conditions. The increased degree of defects for carbon matrix along with applied potentials could be ascribed to the dynamic structural evolution of active Fe–N_3_S_1_ sites, consistent with operando XAS results. The operando spectroscopy measurements indicate that the Fe–N_3_S_1_ sites on highly curved carbon nanosphere undergo geometrically distorted by breaking stretched Fe–S bond to release strain under ORR working conditions. The dynamic optimization of active sites would mitigate overadsorption of key intermediates of *OH and contribute to the durability, thereby dynamically boosting ORR process.

## Conclusions

In summary, local microstrain engineering of asymmetric Fe–N_3_S_1_ sites to optimize electrocatalytic oxygen reduction activity is illustrated. The curved Fe–N_3_S_1_ sites with 1.3% compressed Fe–N bonds and 1.5% stretched Fe–S bond exhibited significantly improved intrinsic ORR activity, selectivity and durability compared to flat Fe–N_3_S_1_. Consequently, the highly strained FeNS-HNS-20 achieved a high half-wave potential of 0.922 V and TOF of 6.2 e^−1^ s^−1^ site^−1^ and negligible decay after 20 k CV cycles. Accordingly, zinc–air batteries assembled with FeNS-HNS-20 exhibited high peak power density of 214 mW cm^−2^, OCV of 1.552 V and superior life span of 2200 cycles. Theoretical calculations revealed the significant role of local microstrain, which would downshift the *d*-band center of central Fe atoms in asymmetric Fe–N_3_S_1_ and accelerate the kinetics of *OH reduction. More importantly, in combination with operando XAS and in situ Raman spectroscopies, the strained Fe–N_3_S_1_ sites were monitored to transform them into Fe–N_3_ sites by breaking stretched Fe–S bond, thereby mitigating the overadsorption of *OH intermediates and contributing to the durability. This work provides a feasible way to further optimize catalytic performance of asymmetric SACs and sheds new light on dynamic evolution of strained asymmetric Fe–N_3_S_1_ sites at atomic precise, paving the way for developing high-performance catalysts with efficient configurations.

## Supplementary Information

Below is the link to the electronic supplementary material.

Supplementary file1 (DOCX 27580 KB)
