# Supplementary material for: Deciphering Local Microstrain-Induced Optimization of Asymmetric Fe Single Atomic Sites for Efficient Oxygen Reduction
Source: Nanomicro Lett. 2025 May 26;17:278. doi: 10.1007/s40820-025-01783-4 (PMC12106294; doi:10.1007/s40820-025-01783-4)
Supplement: Supplementary file 1 — Supplementary file1 (DOCX 27580 KB) [file 40820_2025_1783_MOESM1_ESM.docx]

Supporting Information for

**Deciphering Local Microstrain Induced Optimization of Asymmetric Fe Single Atomic Sites for Efficient Oxygen Reduction**

Peng Zhang^1,2^, Siying Huang^1^, Kuo Chen^1^, Xiaoqi Liu^1^, Yachao Xu^2^, Yongming Chai^1^, Yunqi Liu^1^, and Yuan Pan^1^*

^1^ State Key Laboratory of Heavy Oil Processing, China University of Petroleum (East China), Qingdao, 266580, P. R. China

^2^ School of Materials Science and Engineering, Peking University, Beijing, 100871, P. R. China

*Corresponding author. E-mail: [panyuan@upc.edu.cn](mailto:panyuan@upc.edu.cn) (Yuan Pan)

**S1 Experimental procedures**

**S1.1 Chemicals and materials**

Melamine C_3_H_6_N_6_ (AR, 99%), silica SiO_2_ (AR, 99%, 20 nm)**,** N,N-dimethylformamide C_3_H_7_NO (DMF, AR, 99%), heme chloride C_34_H_32_ClN_4_O_4_Fe (AR, 95%), H_2_SO_4_ (70 wt%), tetraethyl orthosilicate (TEOS, AR, 99%), ethanol (ACS, 99%) , ammonia solution (ACS, 28%) were purchased from Shanghai Aladdin Biochemical Technology Co., Ltd. Coal tar pitch was obtained from Hebei Feitaiyuan Energy Technology Limited Company. All the chemical reagents were used as obtained without further purification.

**S1.2 Characterizations**

The material phase of catalysts was characterized by XRD on D8 advance (Bruker AXS corporation) using Cu Kα radiation. The morphology and micro structure of as-prepare catalysts were monitored by TEM (JEM-2100F) and SEM (JSM-7500F). AFM images were obtained on Shimadzu SPM-9700. Spherical aberration corrected TEM (AC-TEM) was performed on JEM-ARM300F. The specific surface area of catalysts was determined by N_2_ adsorption and desorption isotherms on a 3FLEX instrument (Micrometrics Instrument Ltd.). The metal contents of catalysts were detected by using ICP-OES (Thermo Scientific ICAP 6300). XAFS were carried out at 1W1B station of Beijing Synchrotron Radiation Facility (BSRF). *Operando* XAFS measurements of the Fe K-edge were performed in fluorescence mode using a Lytle detector in the 12B2 Taiwan beamline at SP-8 (Japan). Soft XANES of nitrogen K-edge and sulfur L-edge were conducted at beamlines MCD-A and MCD-B of National Synchrotron Radiation Laboratory (NSRL). *In-situ* Raman spectra was obtained on a Thermo Fisher DXR2 microscope with 533 nm laser excitation at room temperature. Electrochemical measurements were tested using a Princeton potentiostat (Princeton Applied Research).

**S1.3 Electrochemical measurements**

All electrochemical measurements were carried out on pine working stations (Pine instrument company) with standard three-electrode system. The rotating disk electrode (RDE) with a glassy carbon of 5 mm diameter was employed to load the catalyst ink as working electrode. The saturated calomel electrode (SCE) and graphite rod were used as reference and counter electrode, respectively. To prepare the catalysts ink, 5 mg of prepared catalysts were dispersed in 0.5 mL ethanol solutions, containing 10 μL 5 wt% Nafion solutions by sonication for 1 h. The 10 μL of catalysts ink was casted on the electrode for ORR tests and the mass loading was kept at 0.5 mg cm^-2^. The same standard was also used for 20% Pt/C catalysts. Linear sweep voltammetry (LSV) polarization curves were conducted in O_2_-saturated 0.1 M KOH at a sweep rate of 5 mV s^-1^ and a rotating speed of 1600 rpm. The methanol tolerance tests were conducted by chronoamperometric at potential of 0.75 V in 100 mL O_2_-saturated 0.1 M KOH. 5 mL of methanol was injected into the electrolyte at 400 s. The accelerated durability tests (ADT) were carried out by continuously cycling between 1.0 V- 0.75 V at sweep rate of 1000 mV s^-1^ in O_2_-saturated 0.1 M KOH and followed by LSV measurements. The long-term durability tests were performed on a RDE at a rotating speed of 200 rpm in O_2_-saturated 0.1 M KOH under i-t chronoamperometric modes. All the potentials were unified to reversible hydrogen electrode (RHE) based on the equations:

**** (S1)

Where *E(RHE)* and *E(SCE)* are the potential vs RHE and measured potential, respectively. *pH* is the hydrogen ion concentration of the electrolytes. *I* is the measured current and *R*_s_ is the compensated solution resistance.

The hydrogen peroxide yield (H_2_O_2_ %) and electron transfer number (n) were determined based on rotating ring disk electrode (RRDE) tests. The calculated equations were used as follows:

$H_{2}O_{2}(\%)=\frac{200*I_{r}}{I_{r}+N*I_{d}}$ (S2)

$n=\frac{4*I_{d}}{I_{d}+I_{r}/N}$ (S3)

The TOF and site density (SD) of Fe active sites were determined by in situ electrochemical method by means of nitrite absorption followed by reductive stripping developed by Kucernak et al. [S1]. The TOF and SD evaluation of Fe active sites were performed under three-electrode system in 0.5 M acetate buffer electrolyte. The mass loading for catalyst was kept at 0.27 mg cm^-2^. Before the tests, extensive cycling was conducted to remove entrapped gas bubbles within the catalyst layer. The cleaning procedure consisted of extensive cycling, alternating between N_2_-saturated electrolyte at 100 mV/s (20 cycles) and 10 mV/s (10 cycles) and O_2_-saturated electrolyte at 5 mV/s (6 cycles), in the potential window 1.05 to -0.4 V vs. RHE. The above procedure was repeated until stable non changing oxygen reduction behavior and CV under nitrogen were achieved. Then a) record the unpoisoned ORR performance in O_2_-saturated electrolyte at 5 mV/s, 1.0-0.3 V vs. RHE. b) Record a pre-baseline CV in N_2_-saturated electrolyte at potential ranges 1.0-0.3 V vs. RHE, 10 mV/s. c) Record a baseline CV in N_2_-saturated electrolyte at potential ranges 0.4--0.3 V vs. RHE, 10 mV/s. d) Dip RDE into 0.125 M NaNO2 solutions at open circuit potential (OCP) for 5 min, 300 rpm. e) Wash electrode in DI water for 1 min at OCP for 1 min, 300 rpm. f) Wash electrode in the electrolyte for 5 min at OCP, 300 rpm. g) Repeat procedure (e) to remove unspecifically adsorbed nitrite. h) Record ORR of poisoned electrode in O_2_-saturated electrolyte at 5 mV/s, 1.0-0.3 V vs. RHE. i) Record pre-baseline CV of poisoned electrode in N_2_-saturated electrolyte, 1.0-0.3 V vs. RHE, 10 mV/s. j) Record stripping CV in N_2_-saturated electrolyte, 0.4--0.3 V vs. RHE, 10 mV/s. The TOF (s^-1^) and SD (site g^-1^) were calculated based on equations (4) and (5).

$SD=\frac{Q_{strip}}{n_{strip}*F}$ (S4)

$TOF=\frac{n_{strip}*\Delta j_{k}}{Q_{strip}*Lc}$ (S5)

Where *Q_strip_* (C g^-1^) is the excess coulometric charge related to stripping peak, *n_strip_* is the number of electrons by reducing one nitrite per site (*n_strip_*=5), *F* is the Faraday constant of 96485 C mol^-1^. *j_k_* (mA cm^-2^) is the kinetic current density. *j_k_*=(*j_lim_***j*)/(*j_lim_*-*j*), and *∆j_k_=j_k_*(unpoisoned) –*j_k_* (poisoned). *Lc* is the catalysts loadings during in situ experiment, 0.27 mg cm^-2^.

**S145 Zinc-air battery measurements**

A home-built cell was used to evaluated the performance of zinc-air battery of FeNS-HNS-20. The catalyst ink was loaded on carbon paper (1 cm^-2^) with catalysts loadings of 1 mg cm^-2^. A polished zinc plate and carbon paper rolled with gas diffusion layers were used as anode and cathode, respectively. 6 M KOH solutions, containing 0.2 M Zn(OA_C_)_2_ were used as electrolytes. The battery performance was tested on a battery test instrument (Land CT2001A) at room temperature (25 ℃).

**S1.5 Computational details**

The density functional theory (DFT) calculations were conducted by using ab initio simulation package (VASP) [S2, S3]. The generalized gradient approximation in the Perdew-Burke-Ernzerhof (PBE) functional was used to describe the electron exchange and correlation energy [S4]. The frozen-core projector-augmented wave (PAW) method with a cutoff energy of 500 eV was adopted to describe interactions between core electrons and valence electrons [S5]. The empirical DFT-D3 method was applied to describe van der Waals interactions [S6]. The convergence criteria for the energy and force were set to 10^-5^ eV per atom and 0.02 eV/Å, respectively. The reciprocal space was sampled by a grid of (2 × 1 × 1) k-points generated automatically adopting the Monkhorst–Pack method [S7]. The vacuum space was maintained at 15 Å to avoid artificial interactions between perodic images in z direction. The calculation models for strained Fe-N_3_S_1_ sites were built by replacing six carbon atoms of carbon nanotube (diameter of 22 and 180, respectively) with one Fe atom, three N atoms and one S atom. In order to reduce the time required for calculation, the top half of the carbon nanotube was used for calculation, and the bottom two layers of carbon atoms were fixed to simulate the interaction between the bottom half and the top half, while other atoms were allowed to relax. The calculation results showed that compared to the whole carbon nanotube, the atomic displacement rate was less than 1%, which is in line with expectations. The conventional planar Fe-N_3_S_1_ and Fe-N_4_ sites were built by replacing six carbon atoms of flat graphene flake with corresponding atoms. The free energy change of each ORR step was calculated according to the computational hydrogen electrode (CHE) model developed by Nørskov and co-workers [S8, S9]. Based on CHE model, ∆G can be determined using equation:

$\Delta G=\Delta E+\Delta ZPE-T\Delta S$ (S6)

Where ∆*E* is the total energy change obtained from DFT calculations, while ∆ZPE and ∆S are the difference of zero-point energy and entropy, respectively. And T is the temperature of 300 K.

**S2 Supplementary Figures and Tables**


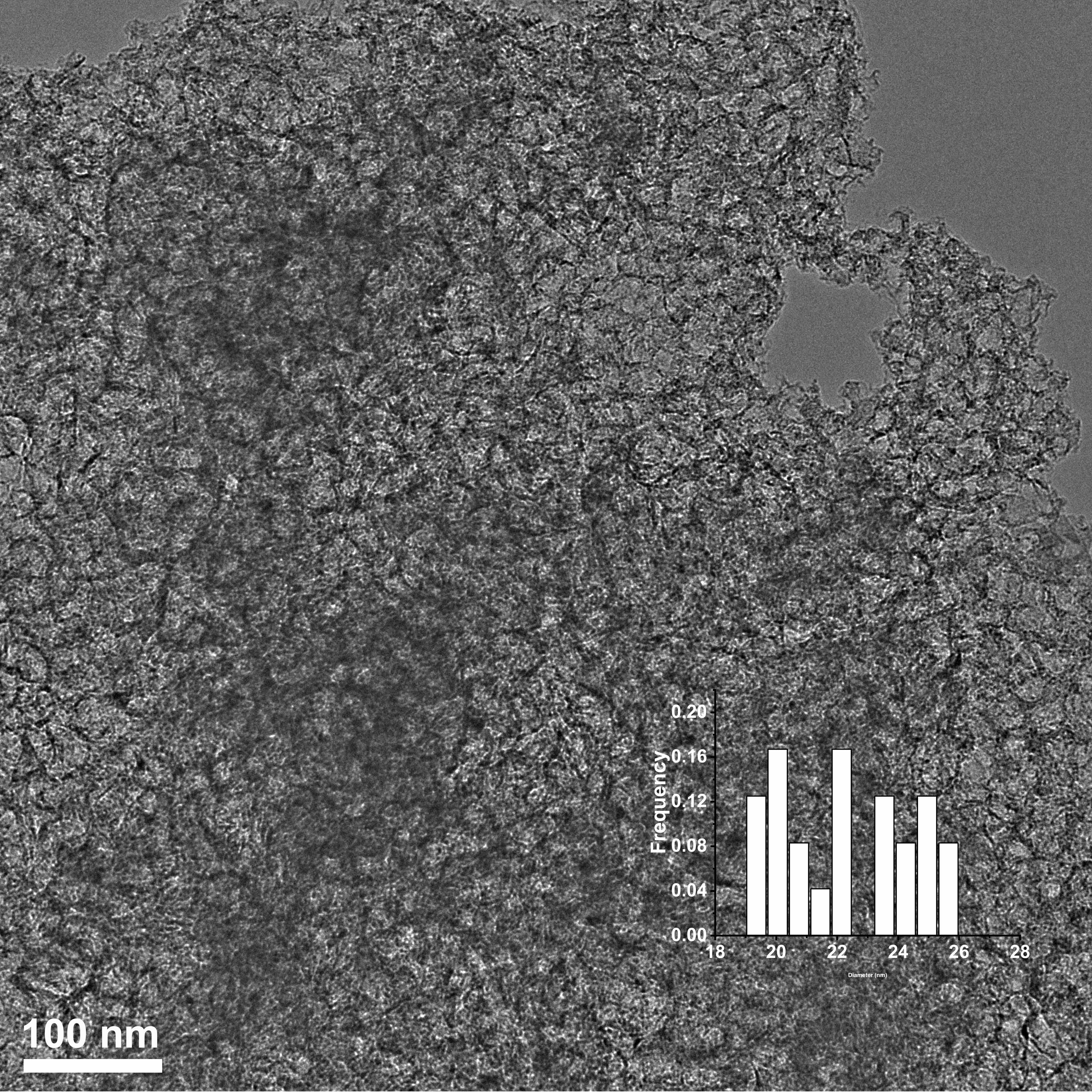


**Fig. S1** TEM images for FeNS-HNS-20. The inset picture shows the calculated size distribution for hollow nanospheres


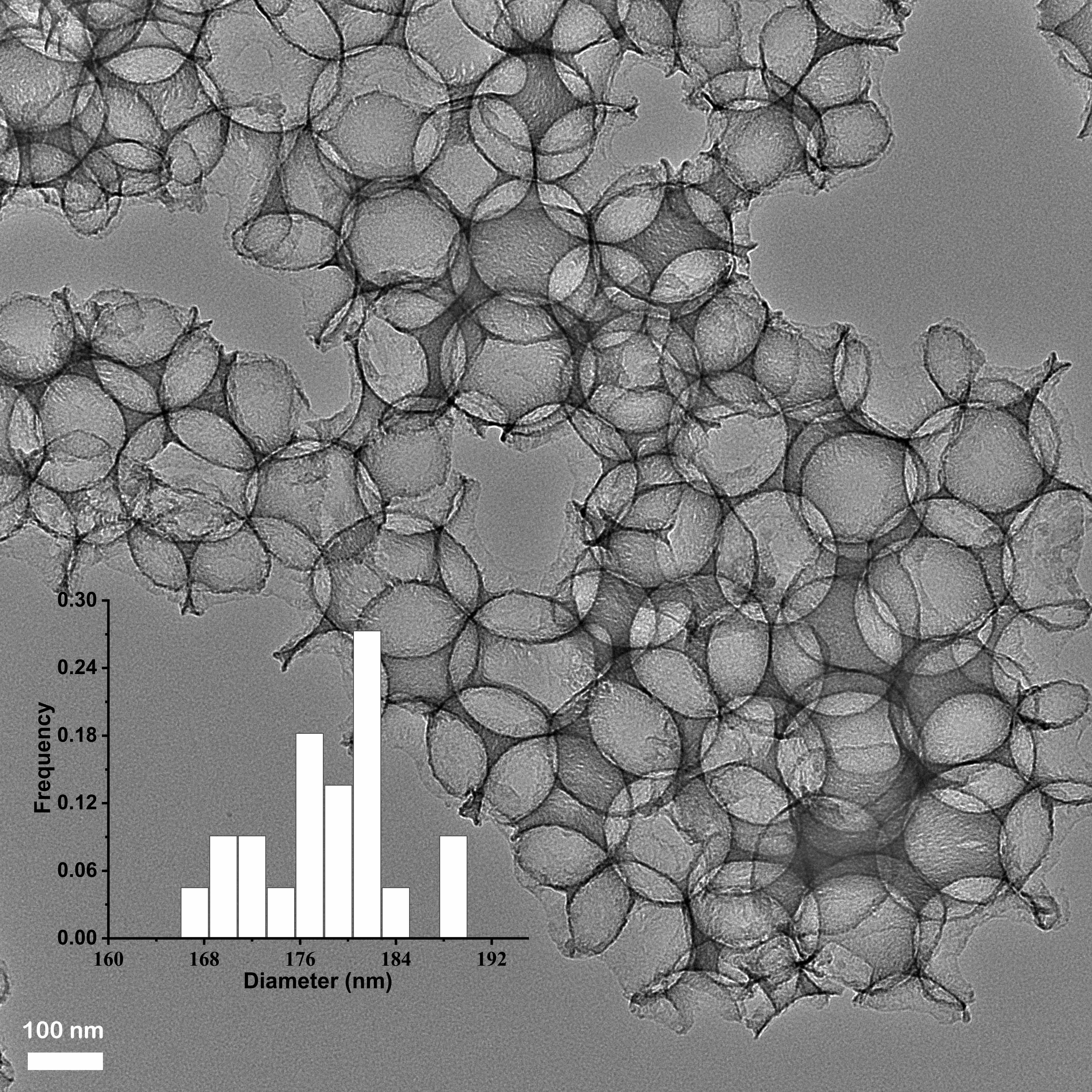


**Fig. S2** TEM images for FeNS-HNS-180. The inset picture shows the calculated size distribution for hollow nanospheres


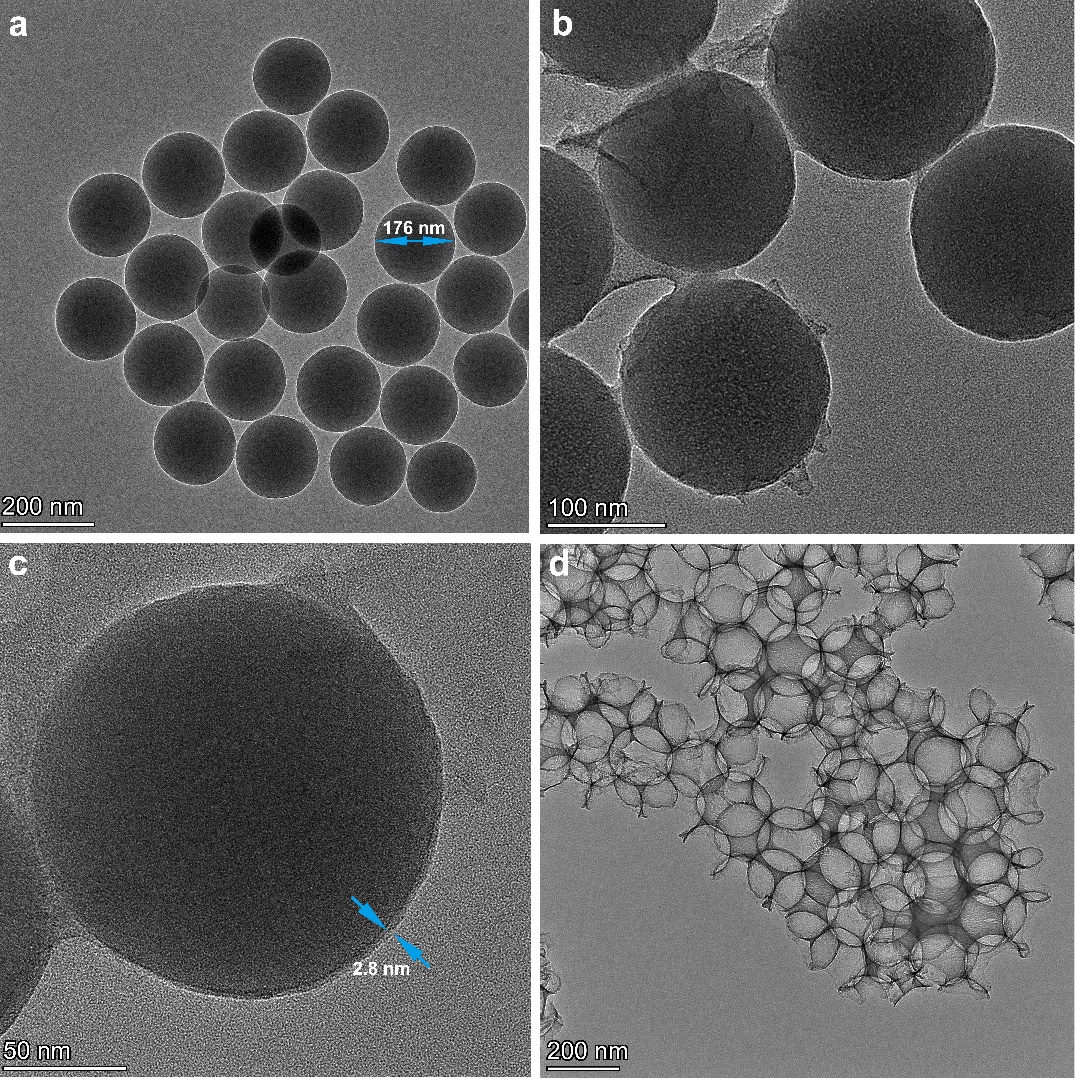


**Fig. S3** Representative preparation procedures for FeNS-HNS-180. (**a**) TEM for SiO_2_ nanospheres. (**b**) TEM for FeNS-HNS-180 before HF etching. (**c**) Magnified TEM for FeNS-HNS-180 with the existence of SiO_2_ (before HF etching), the thickness of carbon layers was 2.8 nm. (**d**) TEM for FeNS-HNS-180 after HF etching


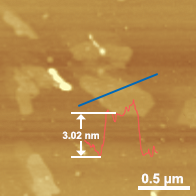


**Fig. S4** AFM images of FeNS-NS


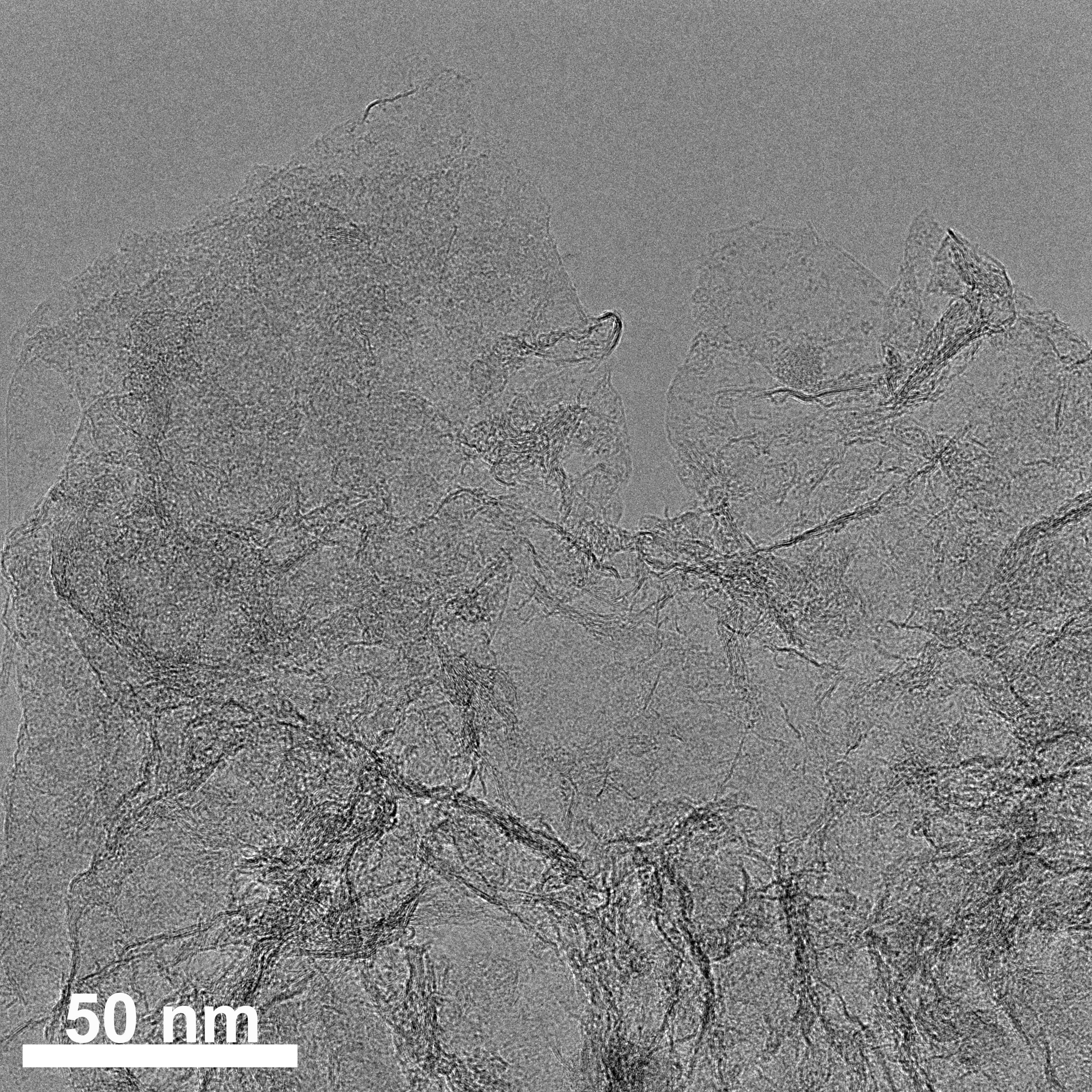


**Fig. S5** TEM images of FeNS-NS


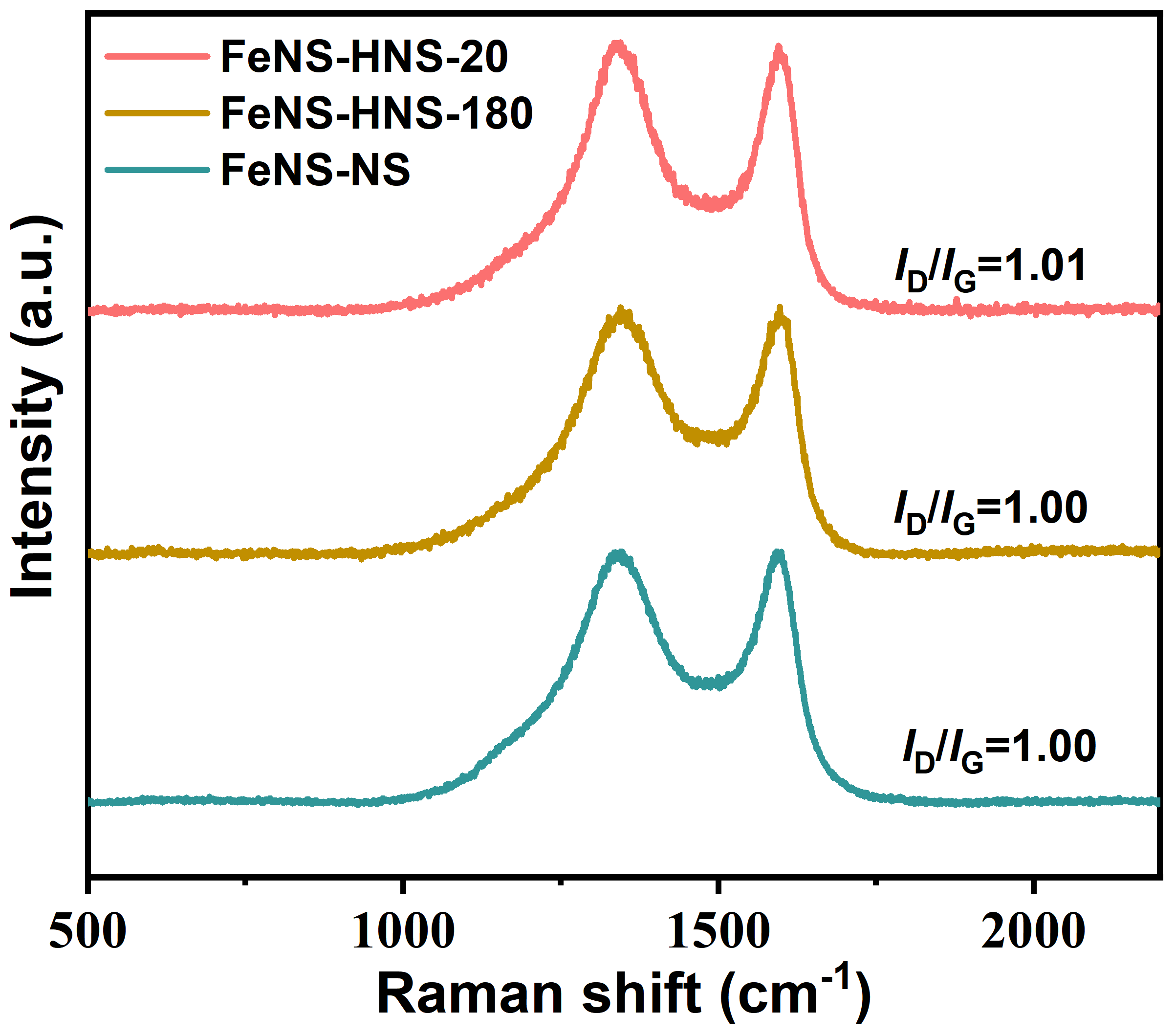


**Fig. S6** Raman spectra for as-prepared catalysts


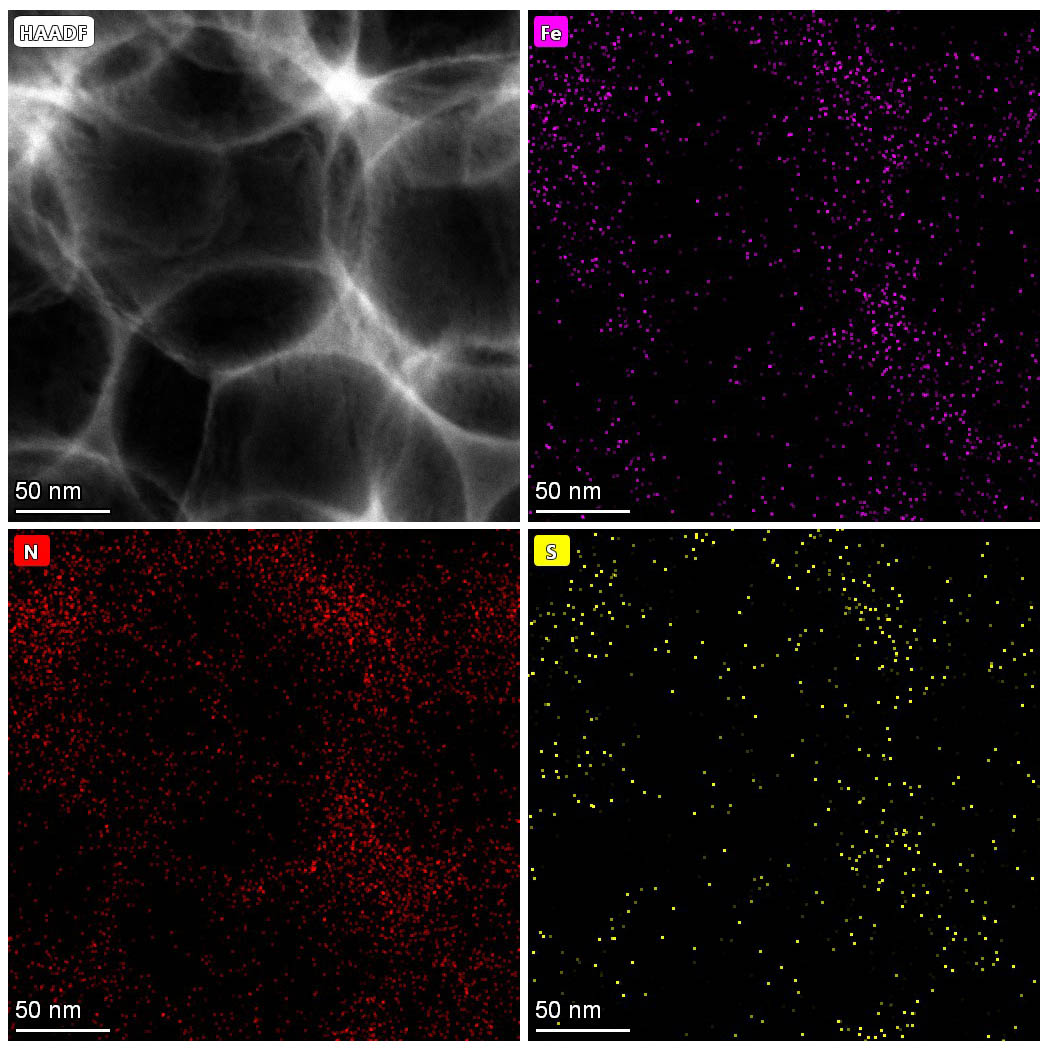


**Fig. S7** HAADF-STEM images of FeNS-HNS-180 and corresponding element mapping of Fe, N and S


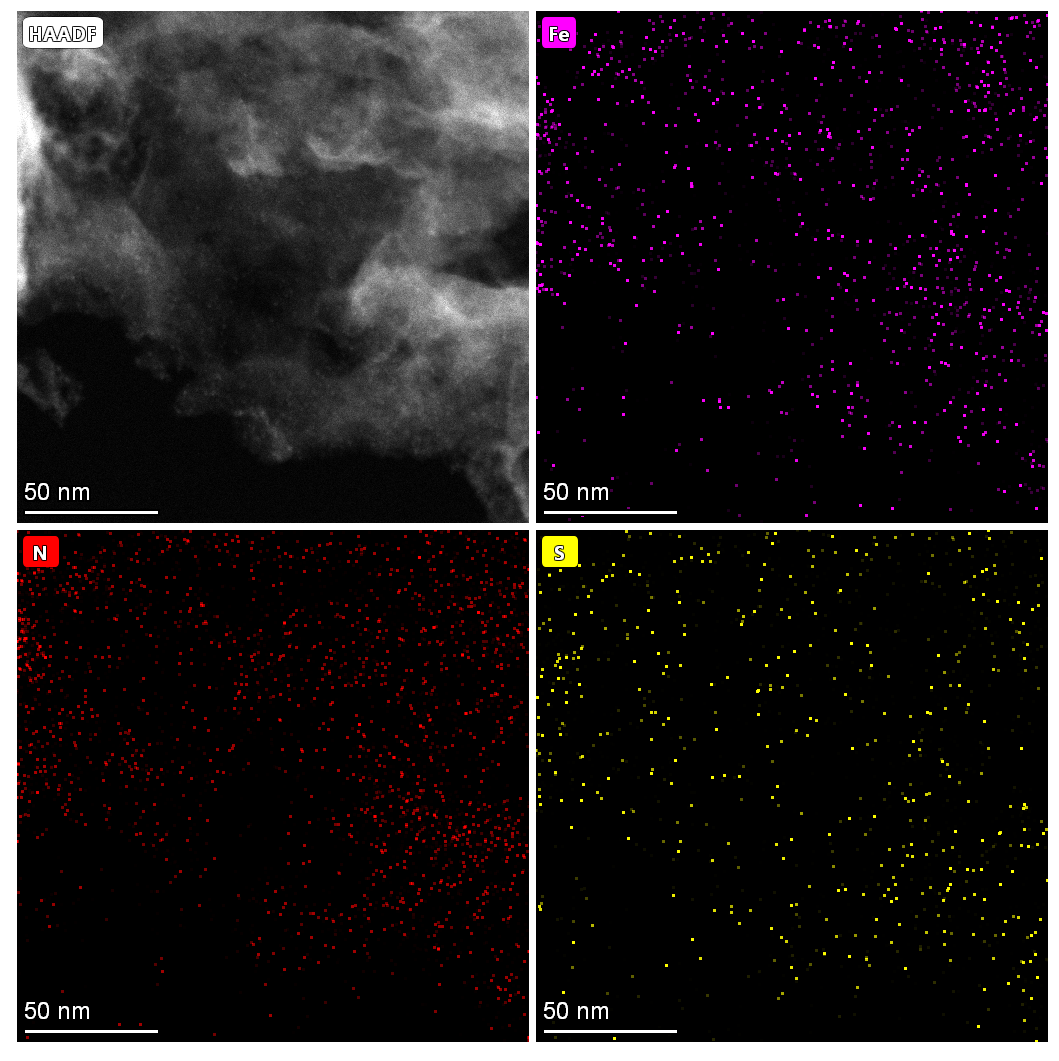


**Fig. S8** HAADF-STEM images of FeNS-NS and corresponding element mapping of Fe, N and S

**
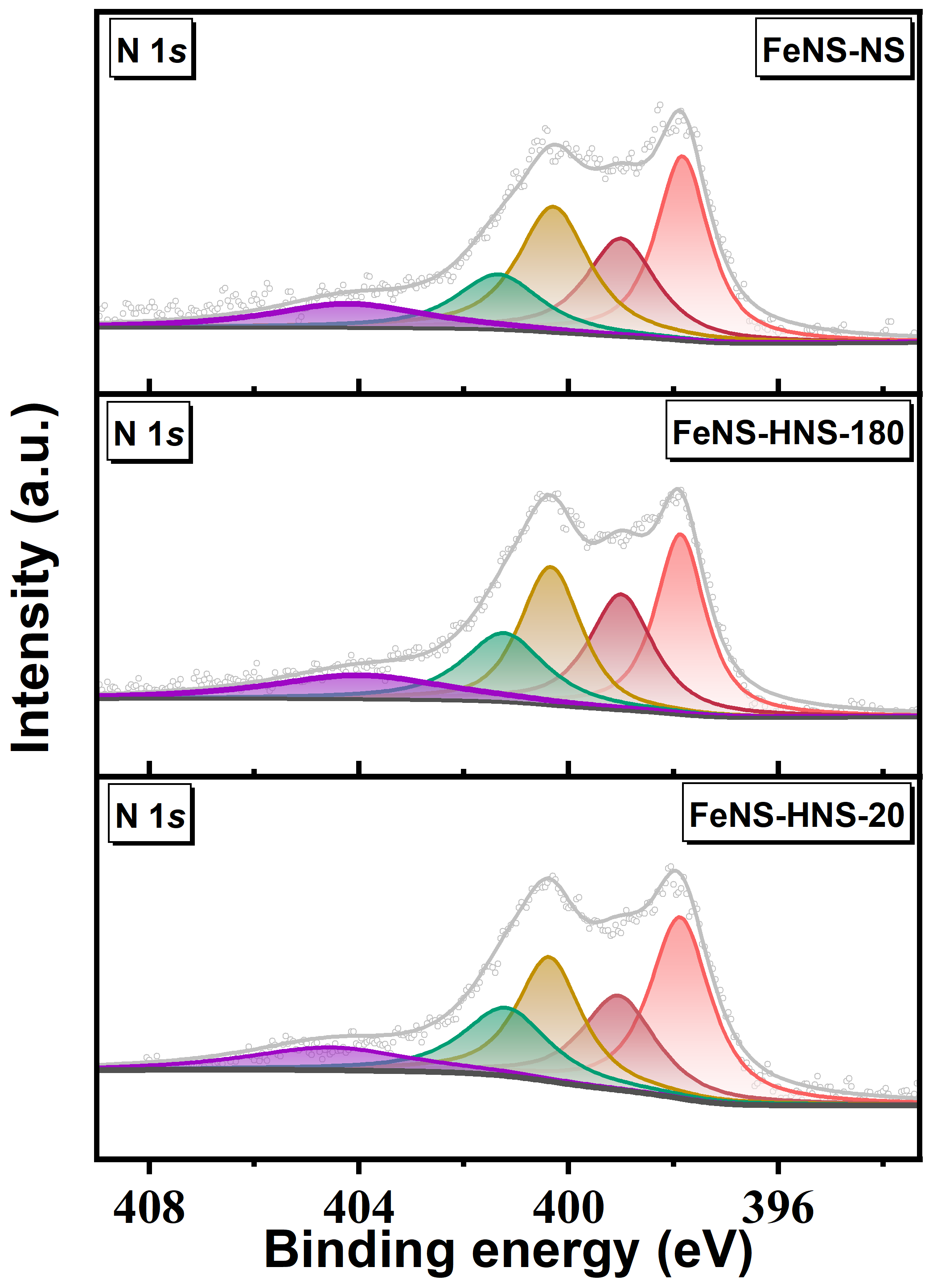
**

**Fig. S9** XPS spectra of N 1*s* for FeNS-NS, FeNS-HNS-180 and FeNS-HNS-20


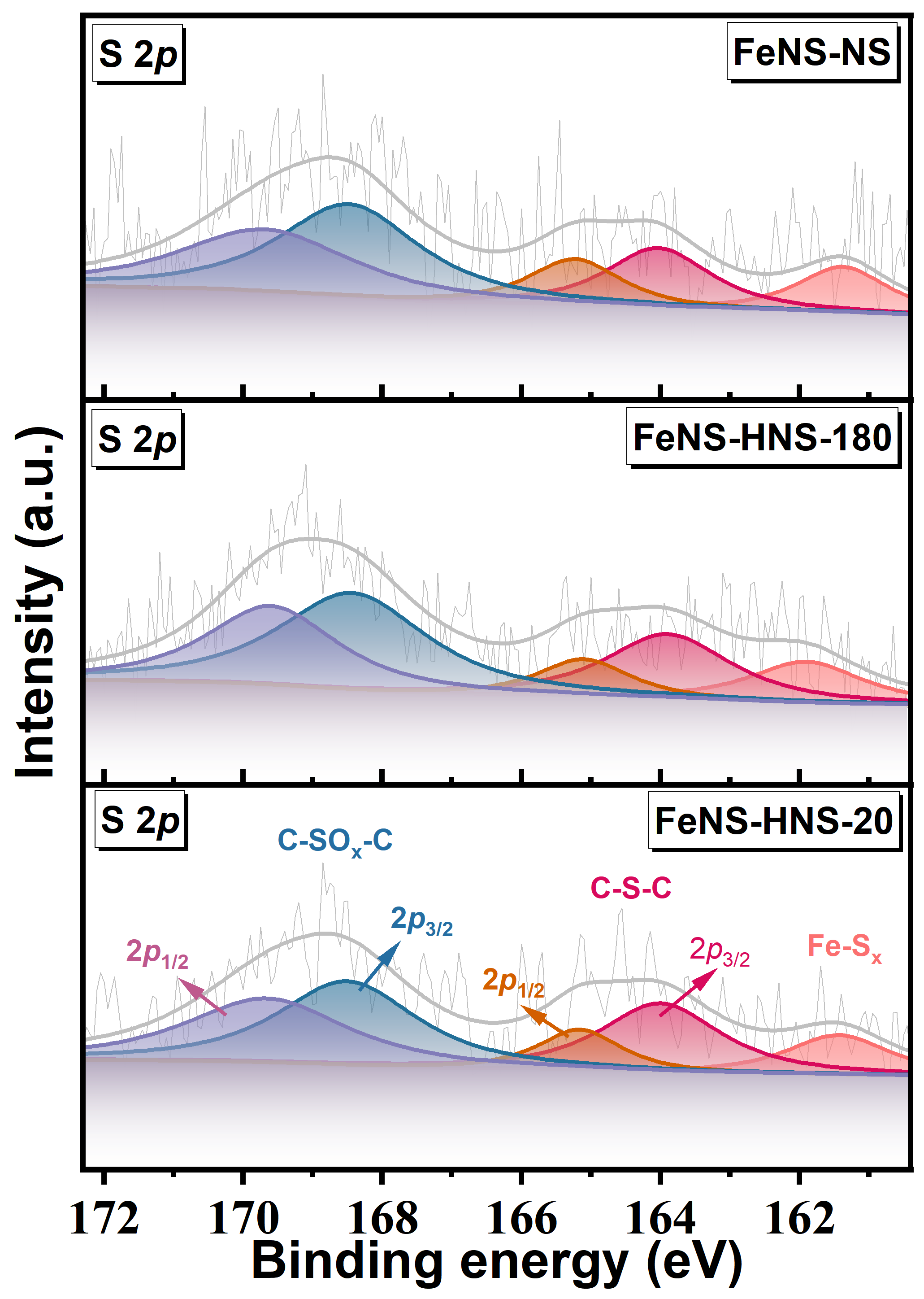


**Fig. S10** XPS spectra of S 2*p* for FeNS-NS, FeNS-HNS-180 and FeNS-HNS-20


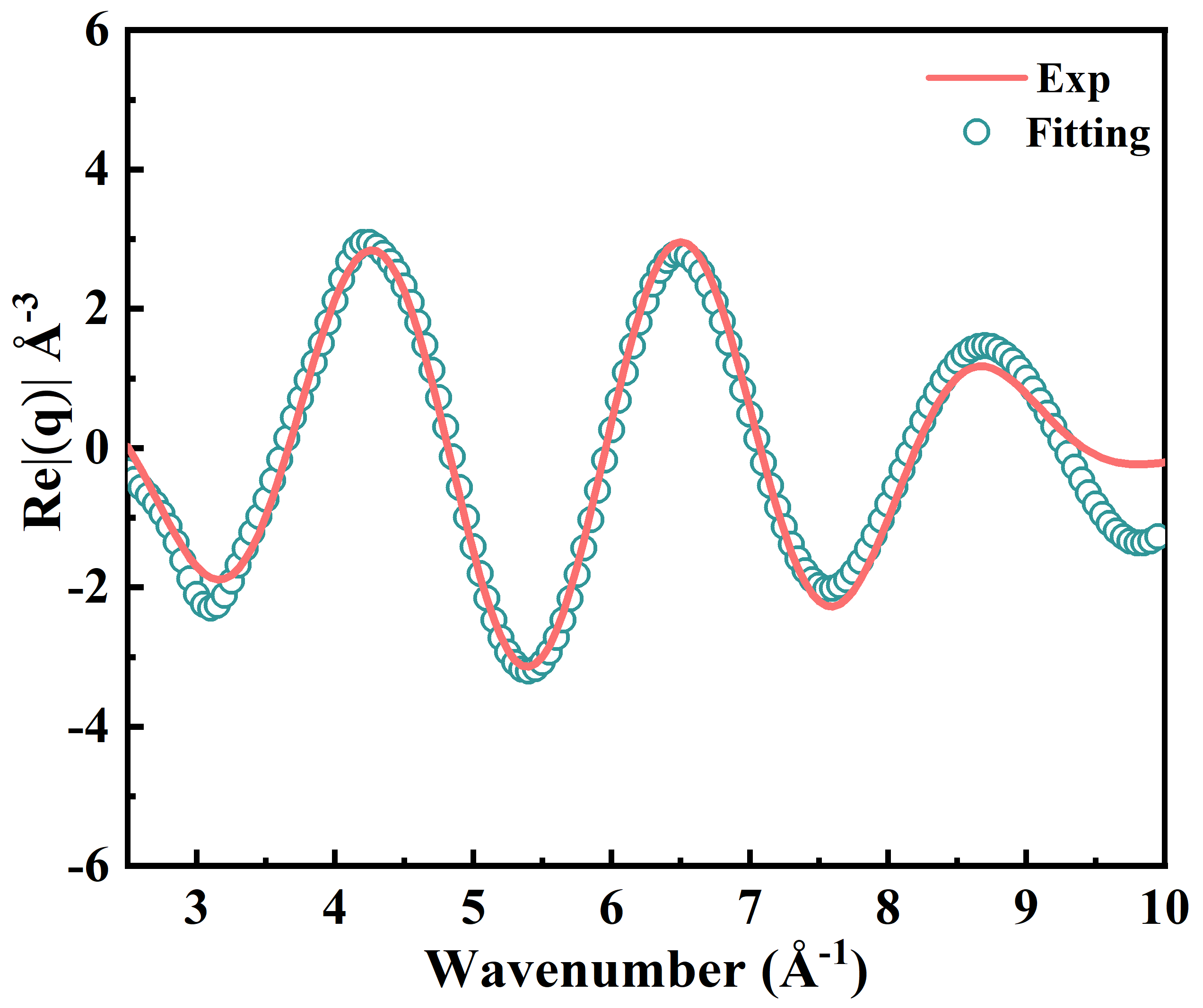


**Fig. S11** EXAFS fitting results of FeNS-HNS-20 in k space


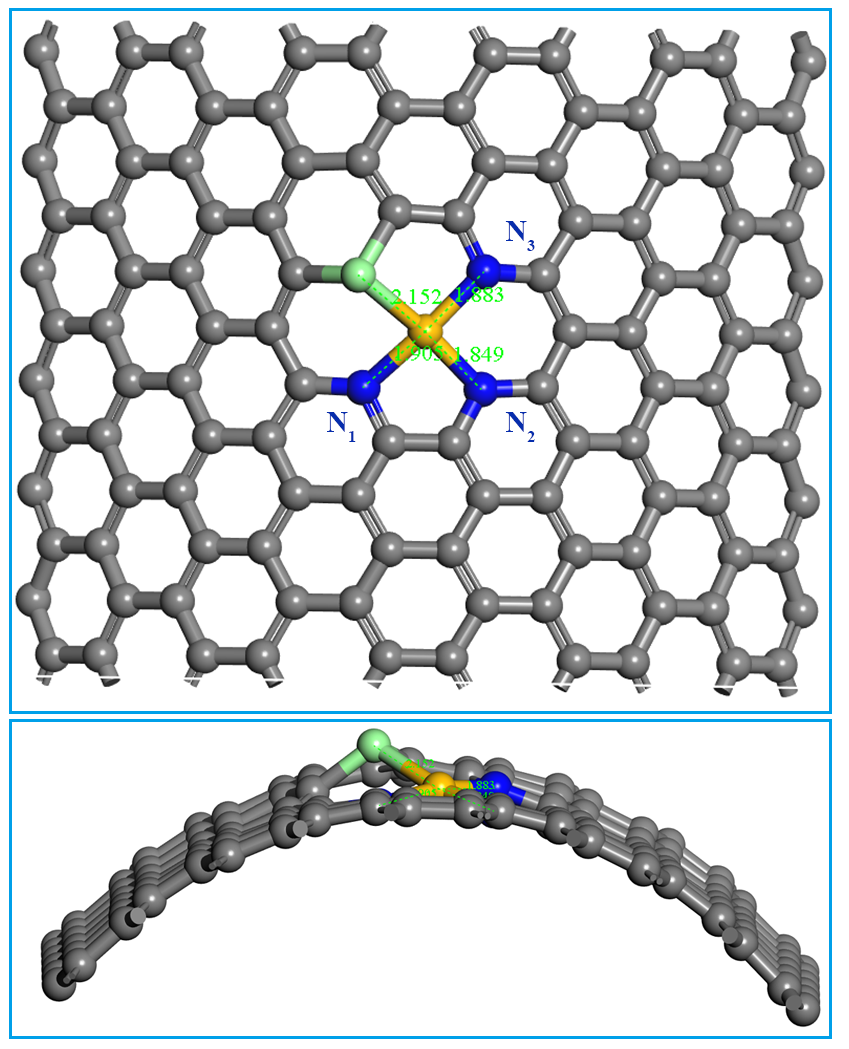


**Fig. S12** Optimized structure for FeNS-HNS-20 with highly strained geometry. The bond length of Fe-S was determined to be 2.152 Å. The bond length of Fe-N_1_, Fe-N_2_ and Fe-N_3_ were determined to be 1.905, 1.849 and 1.883 Å, respectively


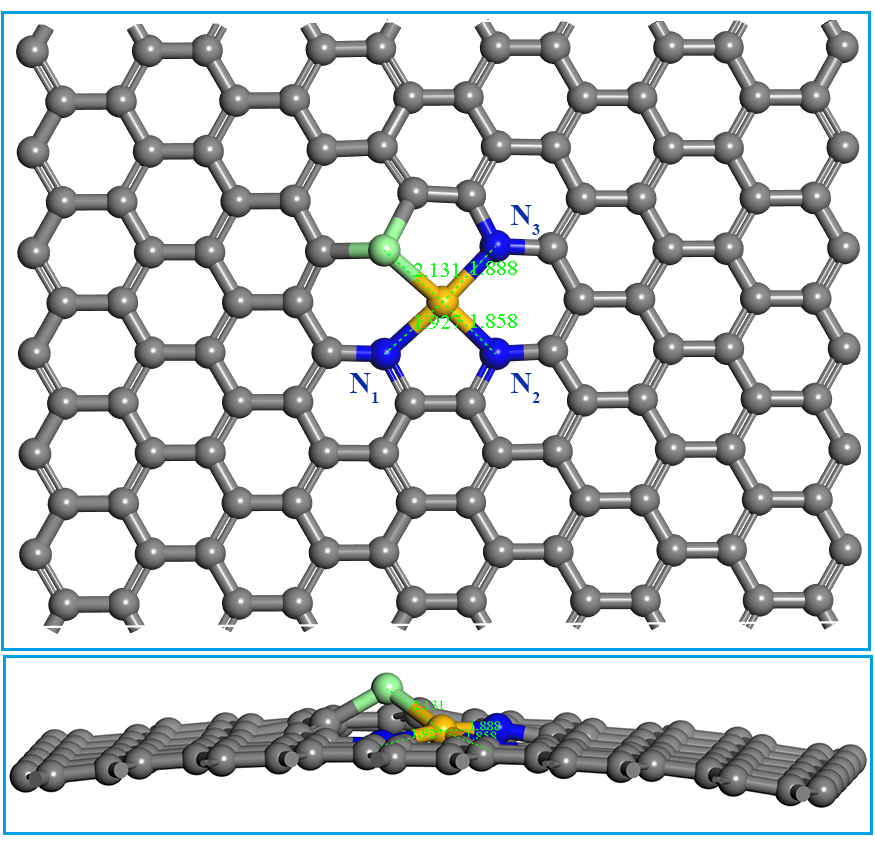


**Fig. S13** Optimized structure for FeNS-HNS-180 with moderately strained geometry. The bond length of Fe-S was determined to be 2.131 Å. The bond length of Fe-N_1_, Fe-N_2_ and Fe-N_3_ were determined to be 1.927, 1.858 and 1.888 Å, respectively


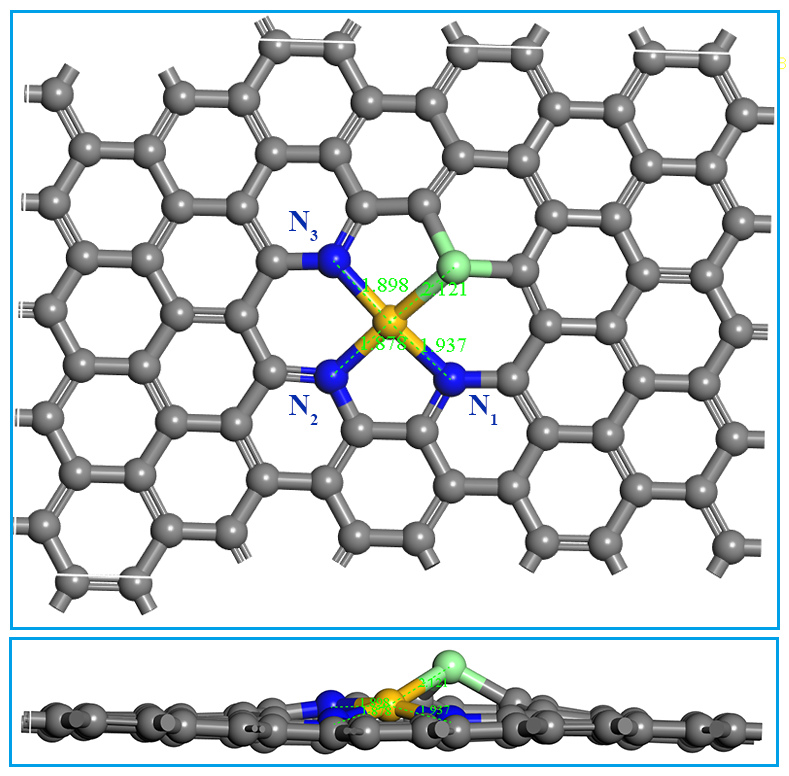


**Fig. S14** Optimized structure for FeNS-NS with flat geometry. The bond length of Fe-S was determined to be 2.121 Å. The bond length of Fe-N_1_, Fe-N_2_ and Fe-N_3_ were determined to be 1.937, 1.878 and 1.898 Å, respectively


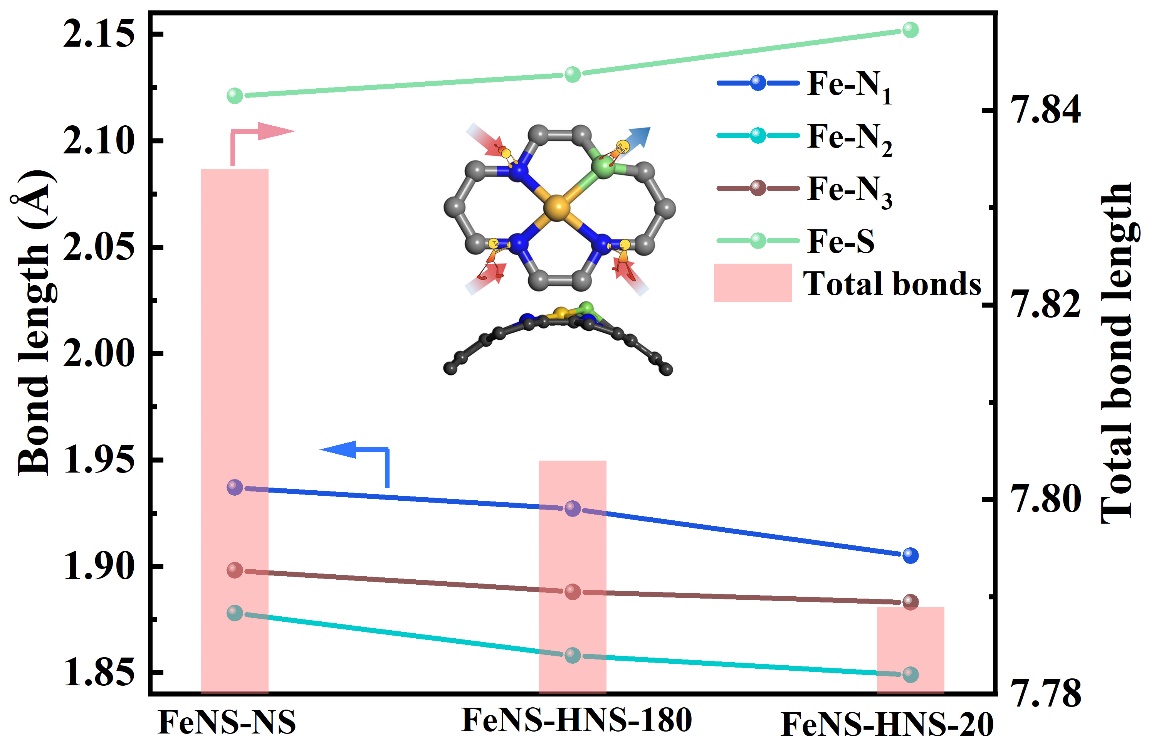


**Fig. S15** Summarized Fe-N, Fe-S and total bond length of Fe-N_3_S_1_ sites with different curvatures based on optimized structure models. All the Fe-N bonds show decreased bonds length with increasing curvature, while the Fe-S bonds demonstrate an increased bond length. That is, compressive Fe-N bonds and tensile Fe-S bonds were achieved as a result of substrate curvature bending. Compared to flat FeNS-NS, the Fe-N bonds in FeNS-HNS-180 are compressed by 0.7%, while the Fe-S bond is elongated by 0.5%, suggesting multilevel local microstrain is created on asymmetric Fe-N_3_S_1_ sites by curved structure


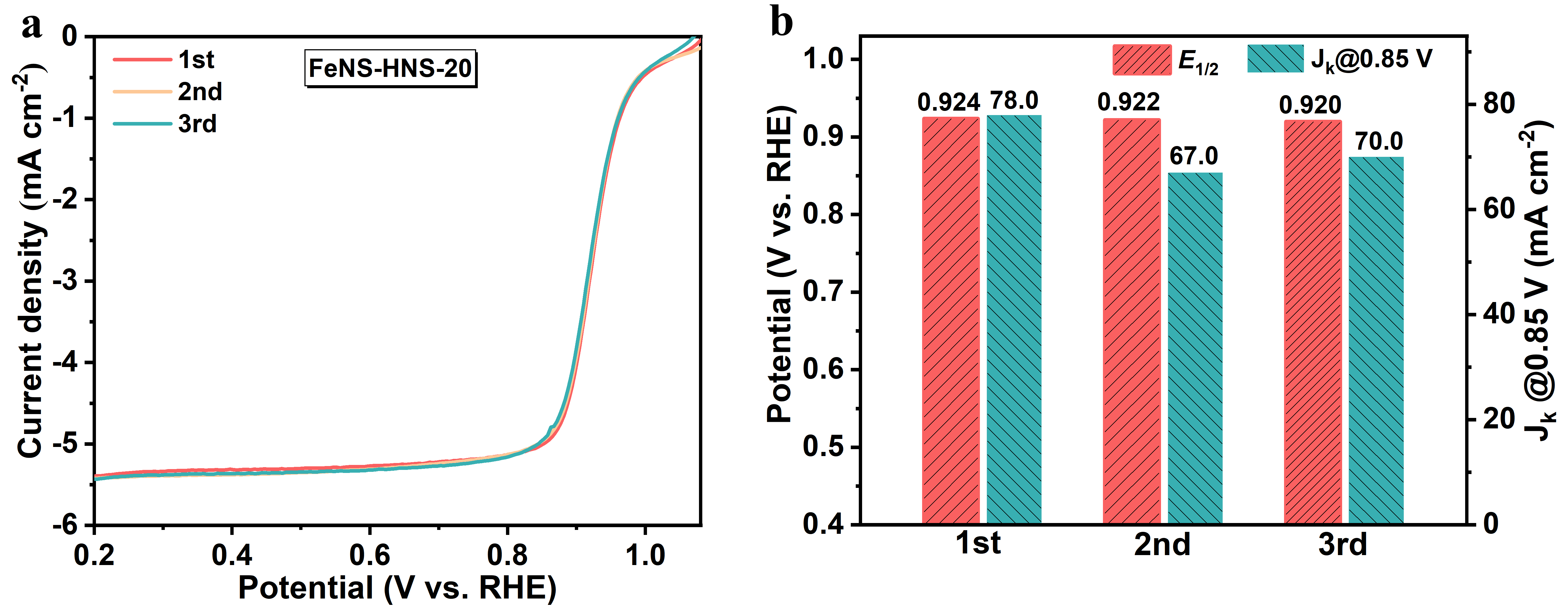


**Fig. S16** Independent experiments for FeNS-HNS-20. (**a**) LSV curves, (**b**) Half-wave potential and kinetic current density at 0.85 V


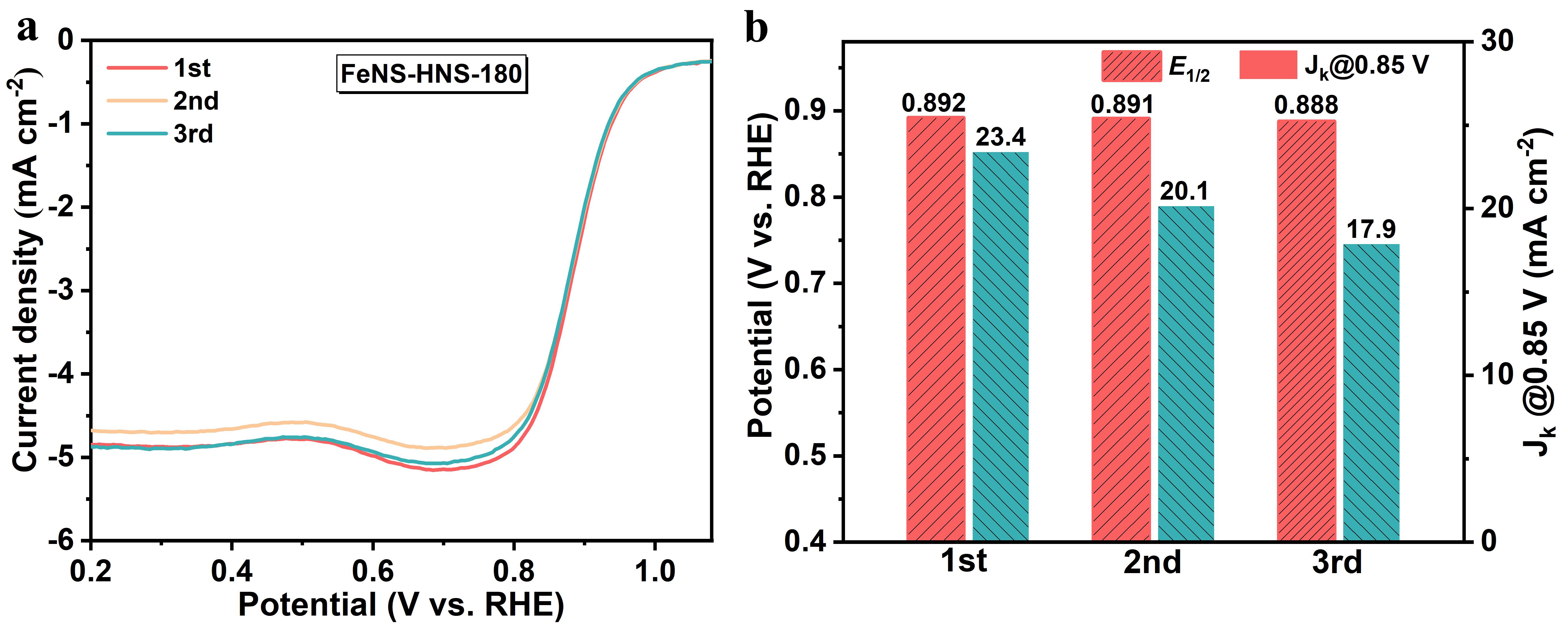


**Fig. S17** Independent experiments for FeNS-HNS-180. (**a**) LSV curves, (**b**) Half-wave potential and kinetic current density at 0.85 V


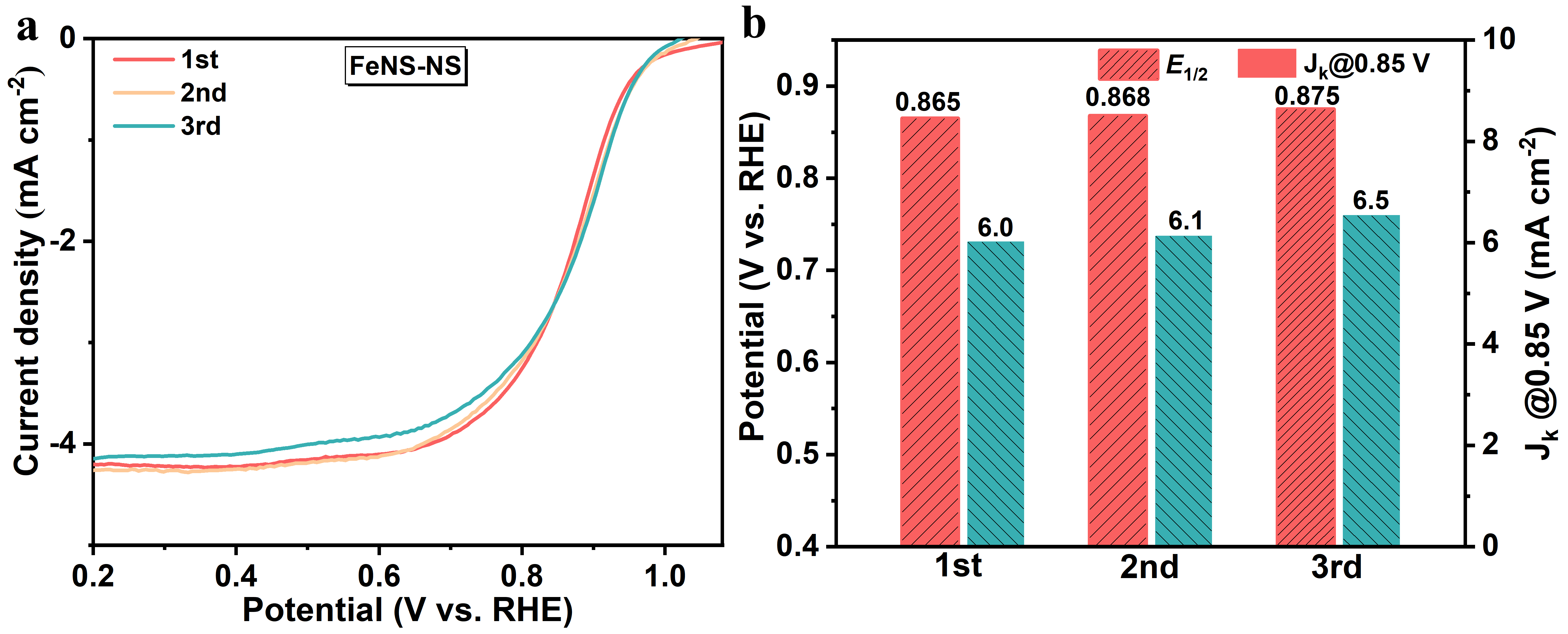


**Fig. S18** Independent experiments for FeNS-NS. (**a**) LSV curves, (**b**) Half-wave potential and kinetic current density at 0.85 V


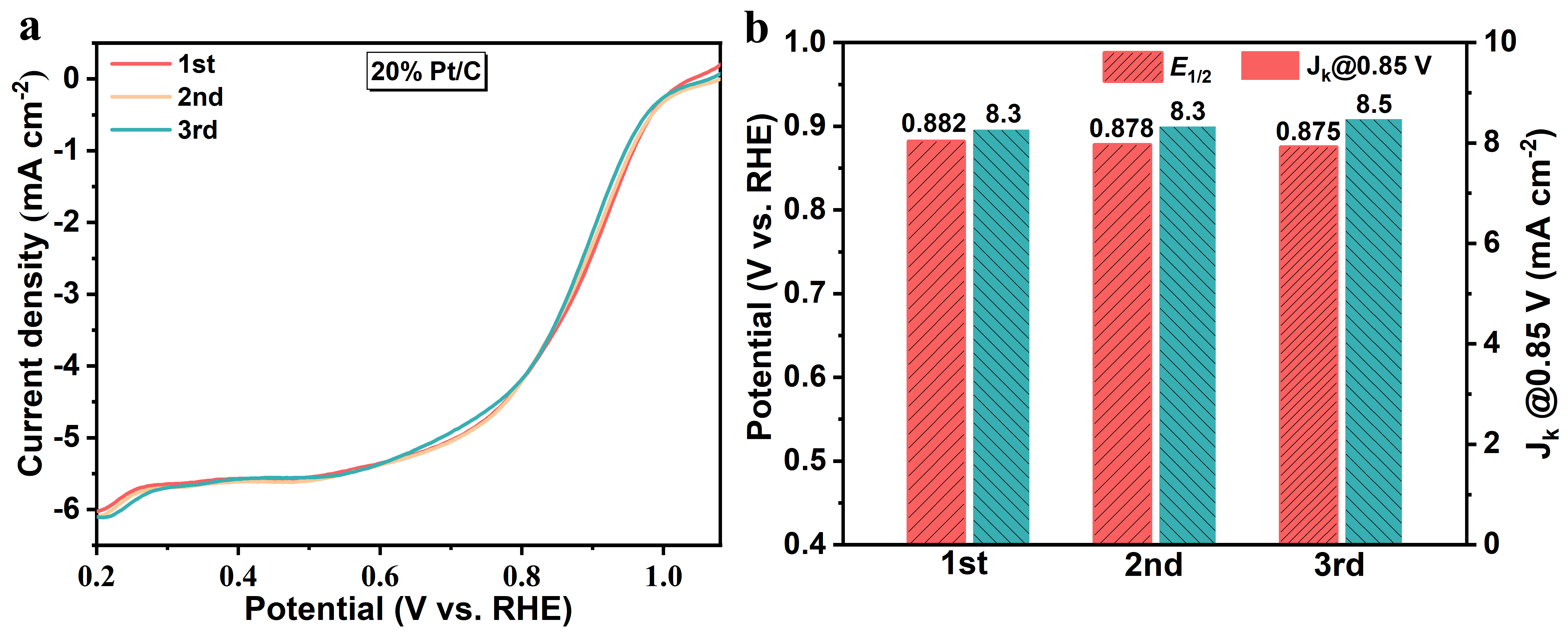


**Fig. S19** Independent experiments for 20% Pt/C. (**a**) LSV curves, (**b**) Half-wave potential and kinetic current density at 0.85 V


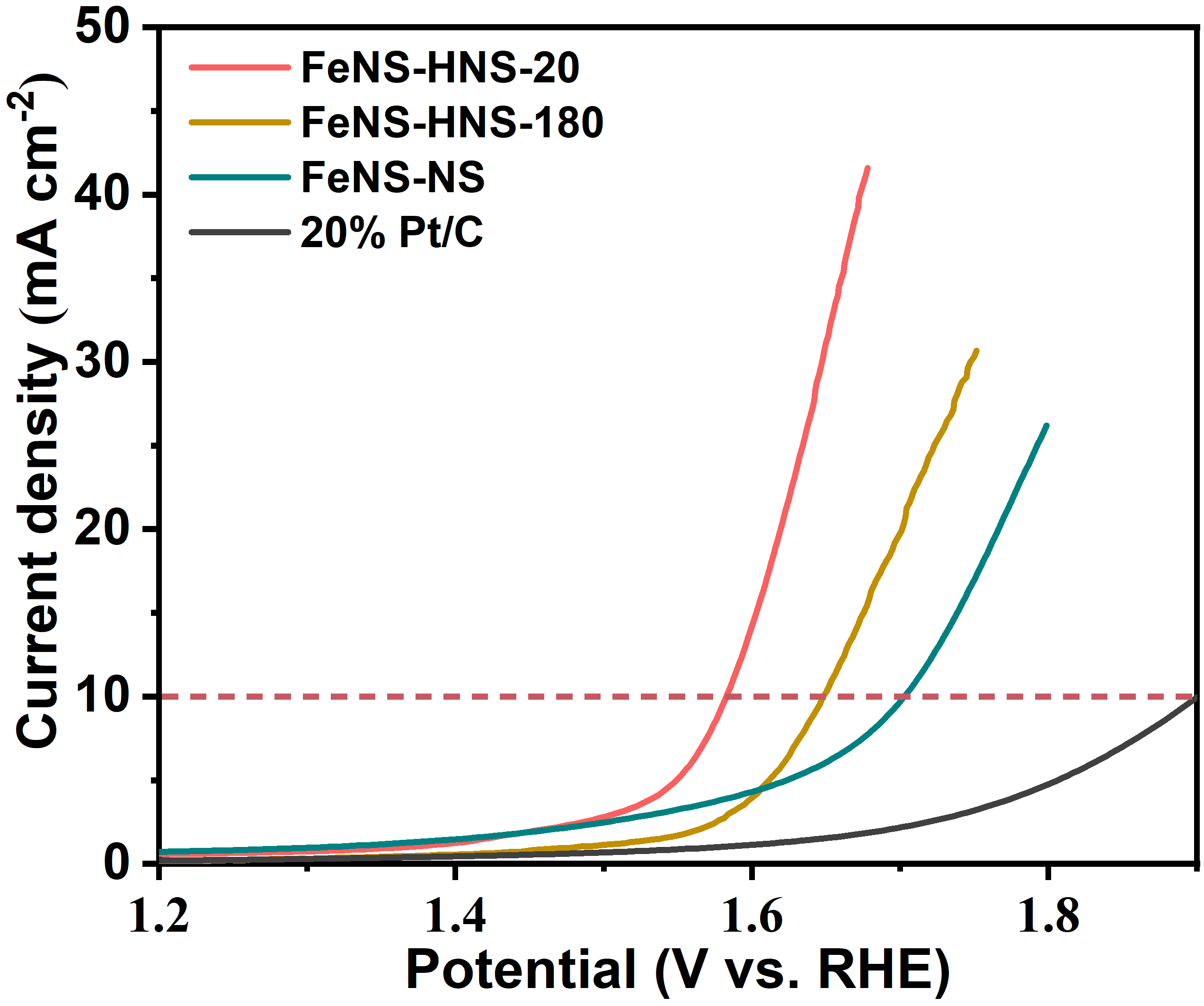


**Fig. S20** OER performance of as-prepared catalysts and commercial Pt/C


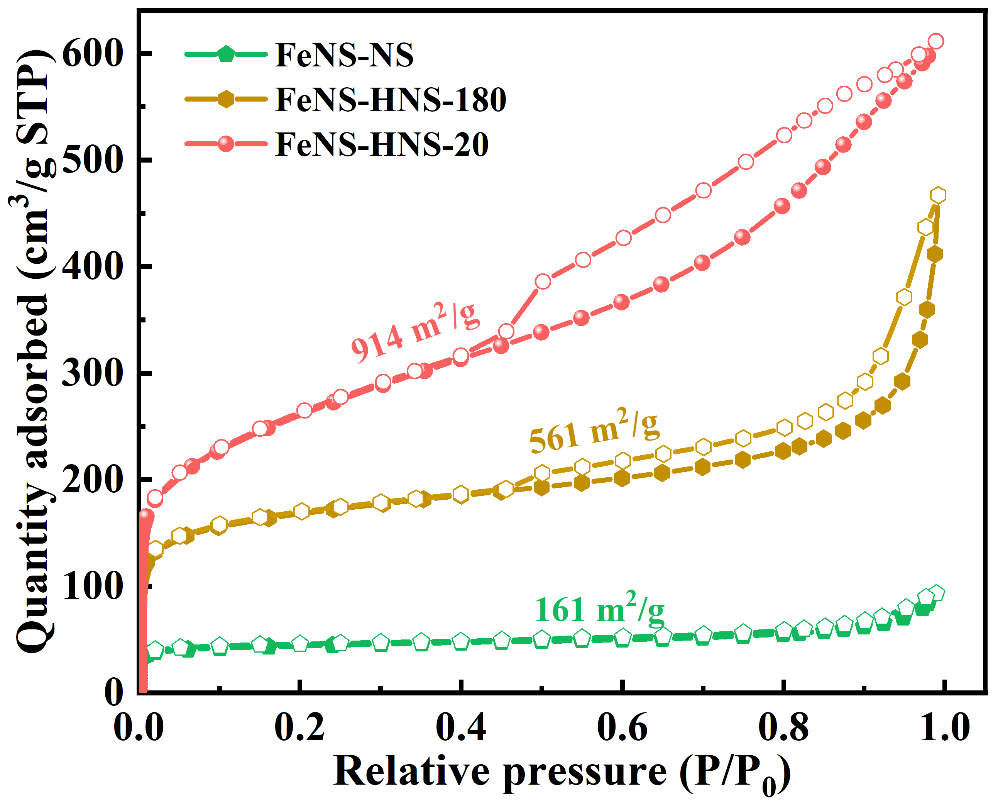


**Fig. S21** Nitrogen adsorption and desorption isotherms of FeNS-HNS-20, FeNS-HNS-180 and FeNS-NS, respectively


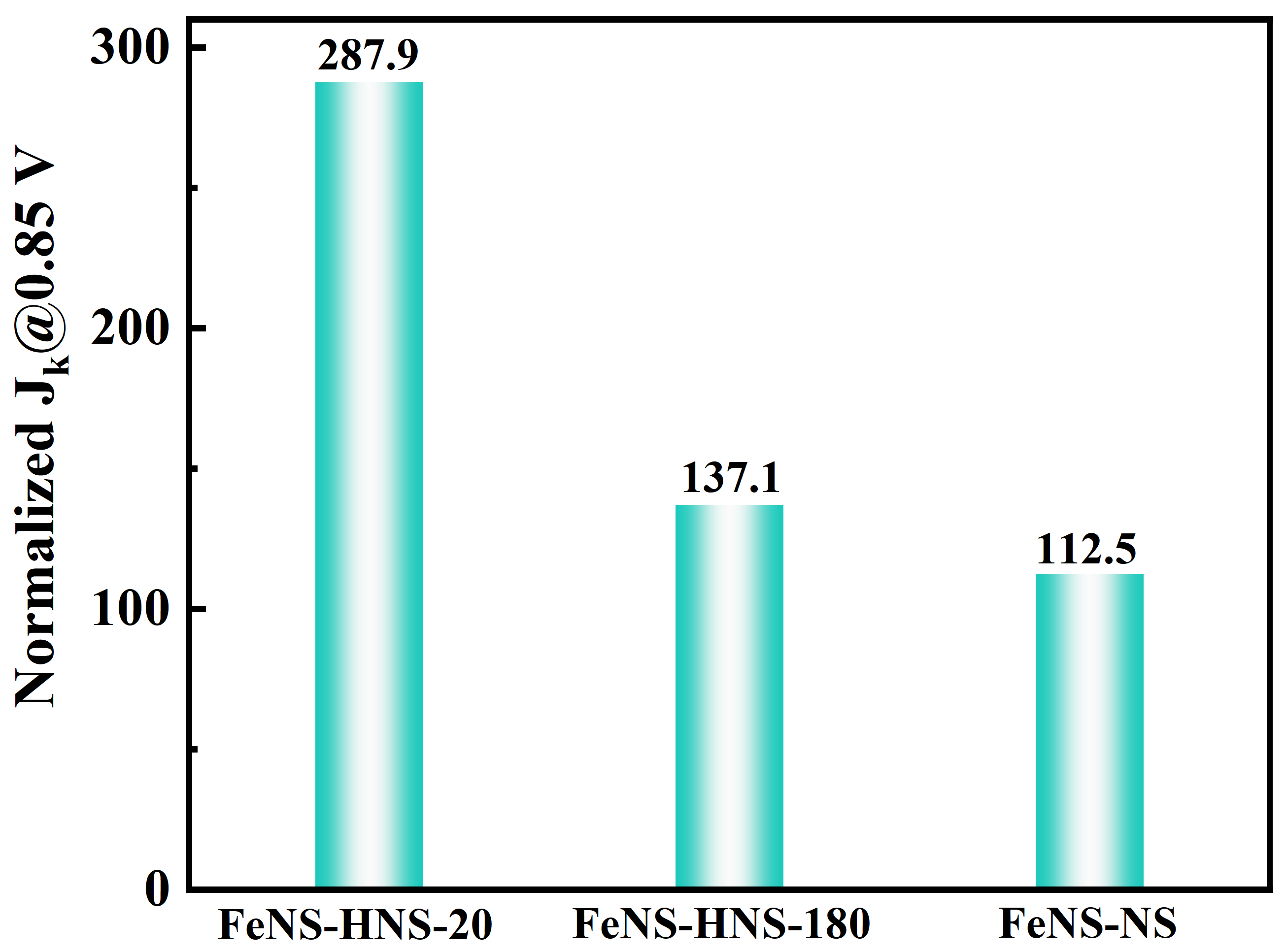


**Fig. S22** Kinetic current density (mA cm^-2^) of catalysts at 0.85 V normalized by specific surface area (m^2^/g) and Fe contents (mg)


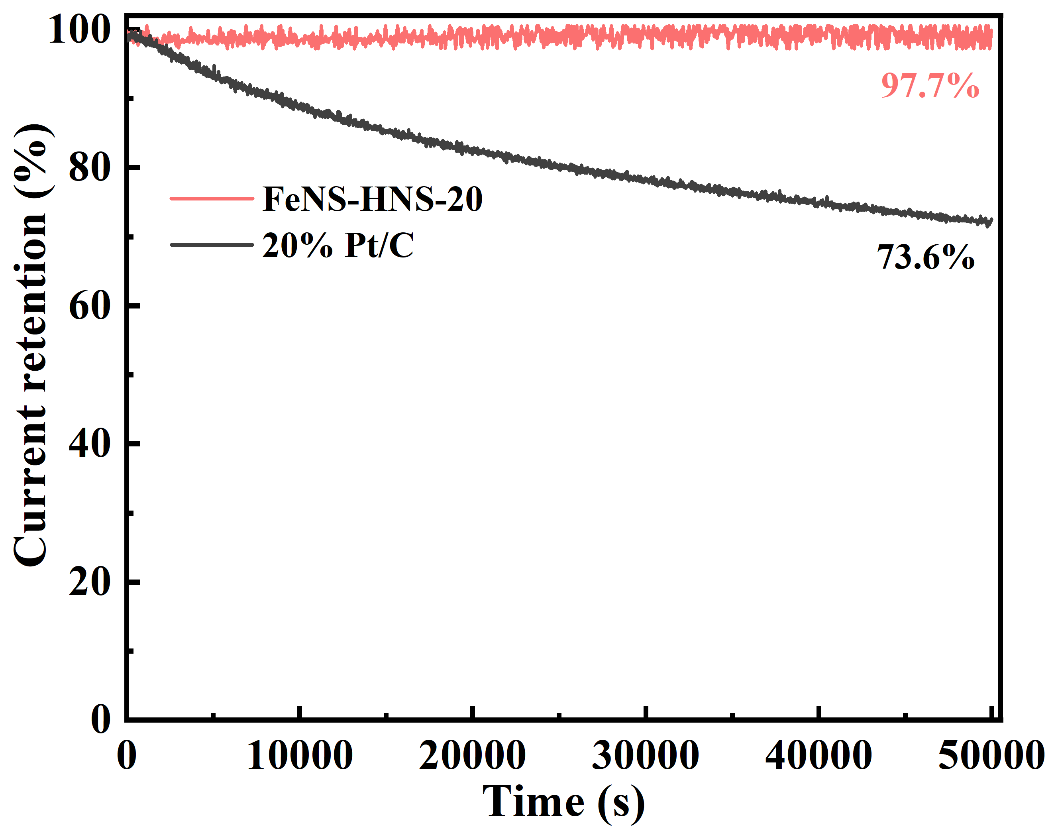


**Fig. S23** Long-term i-t chronoamperometric tests of FeNS-HNS-20 and 20% Pt/C benchmarks


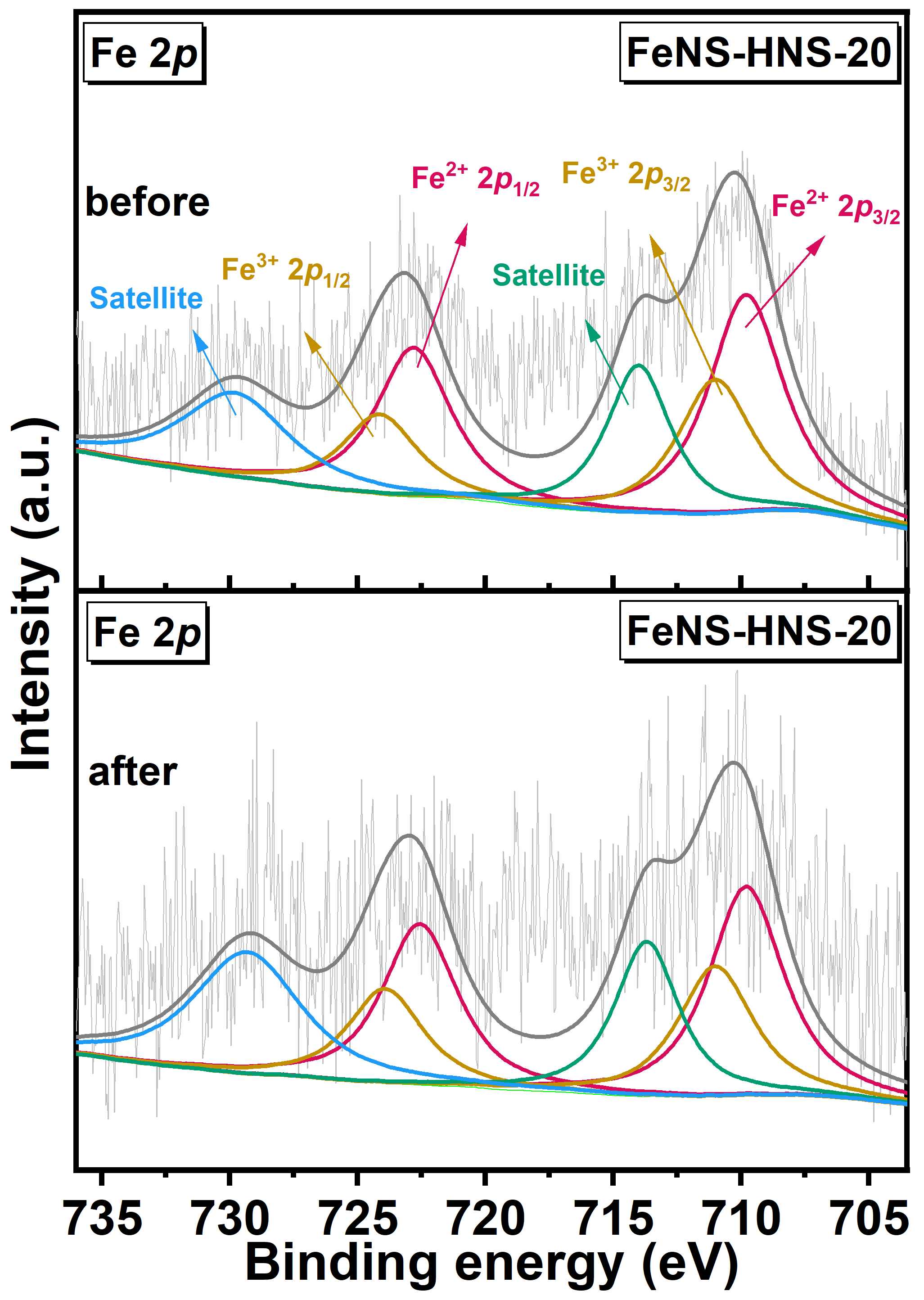


**Fig. S24** XPS spectra of Fe 2*p* for FeNS-HNS-20 before and after i-t stability tests


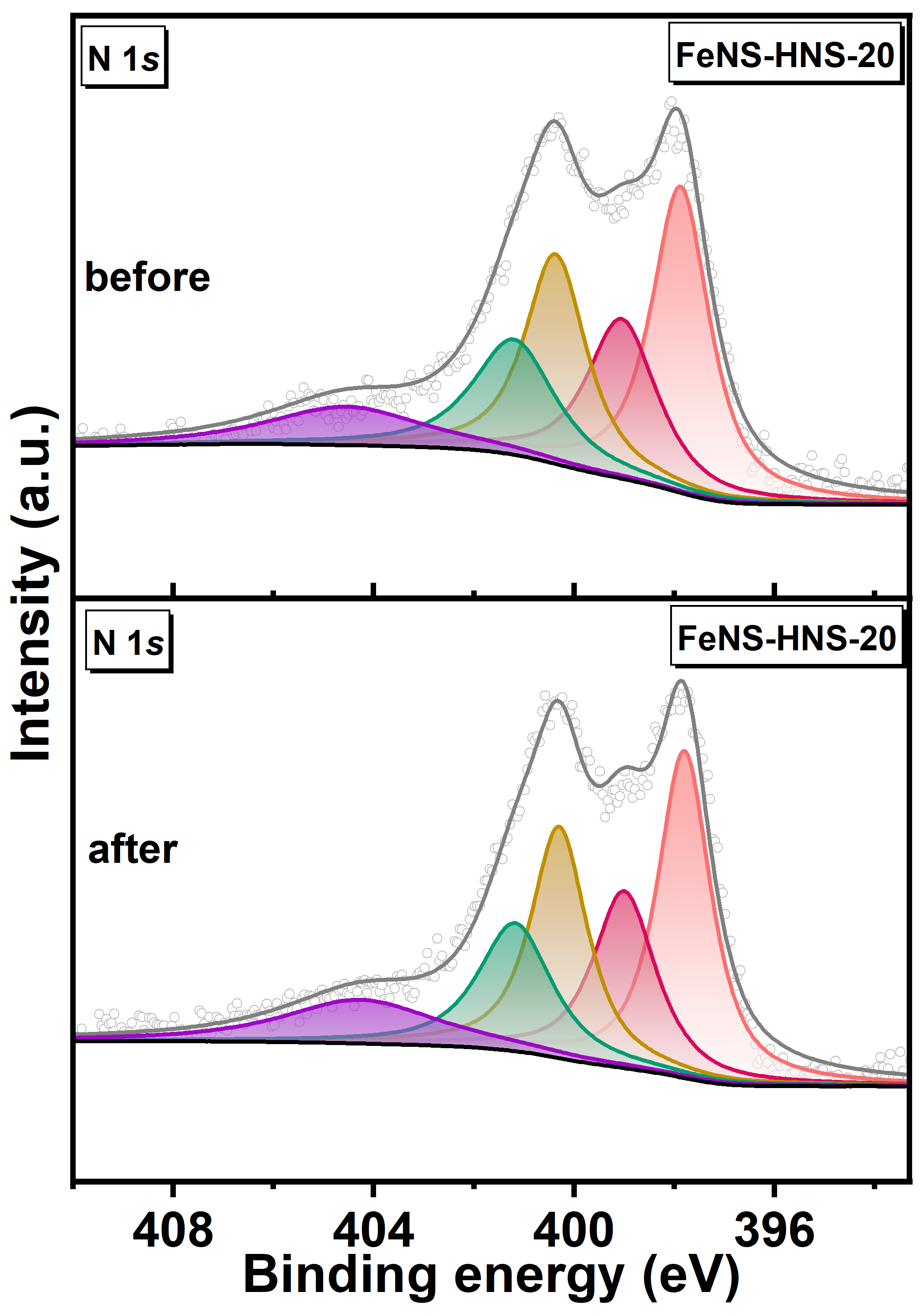


**Fig. S25** XPS spectra of N 1*s* for FeNS-HNS-20 before and after i-t stability tests


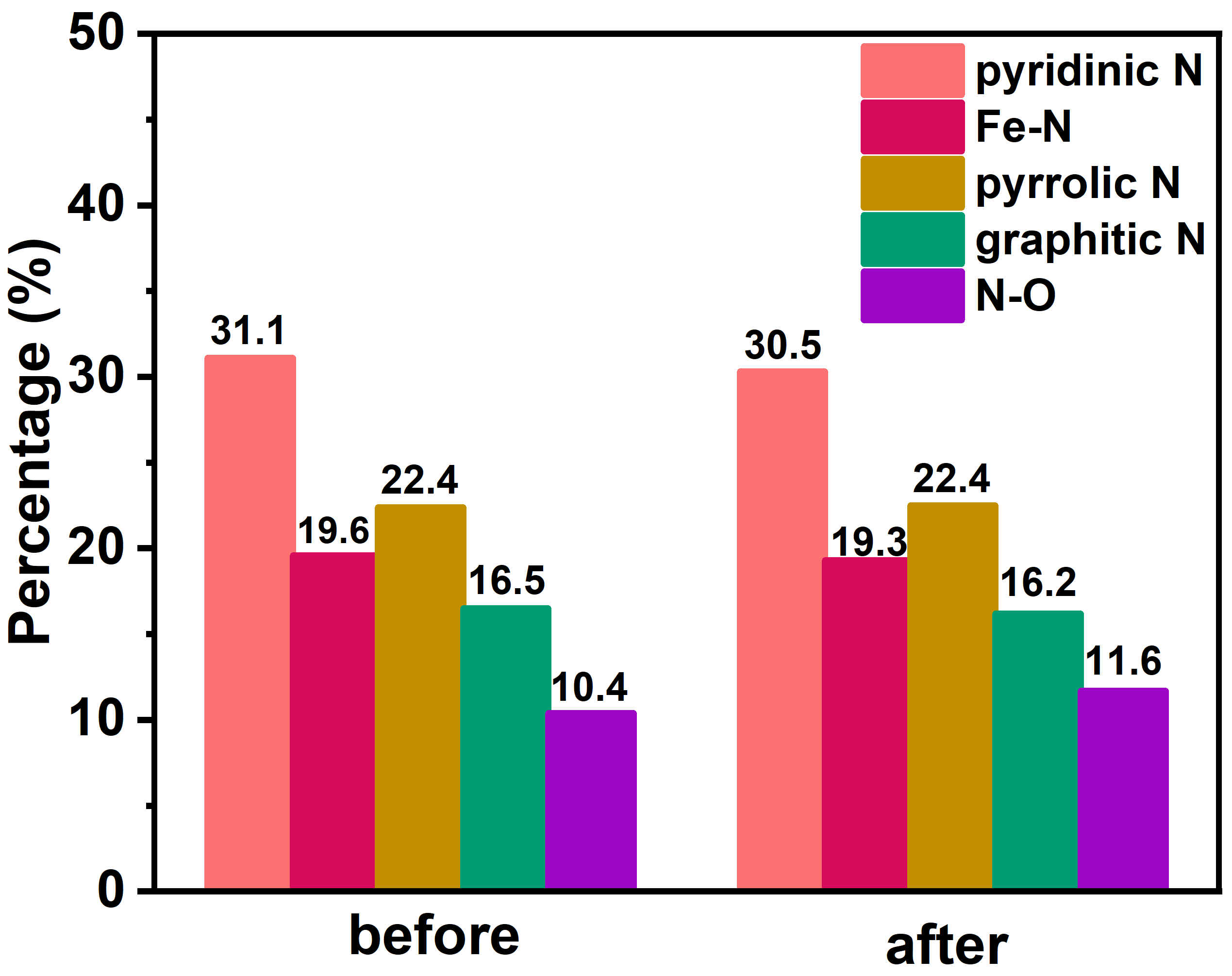


**Fig. S26** N proportions for FeNS-HNS-20 before and after i-t stability tests


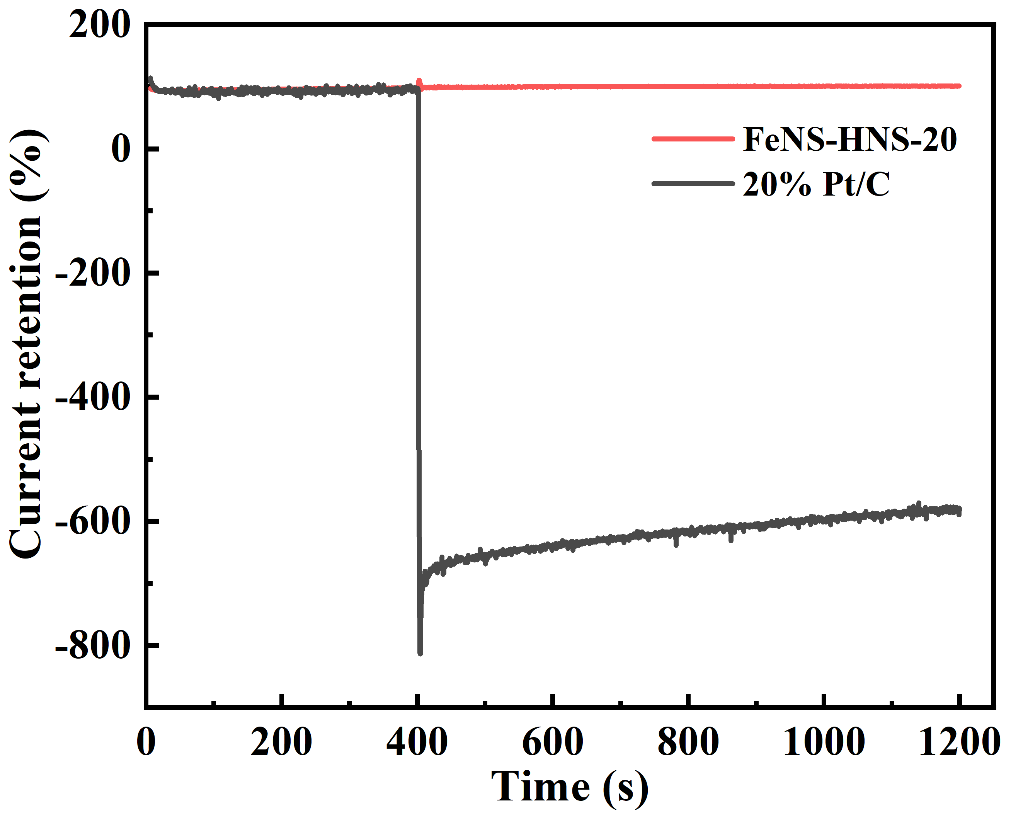


**Fig. S27** Methanol tolerance tests of FeNS-HNS-20 and 20% Pt/C benchmarks


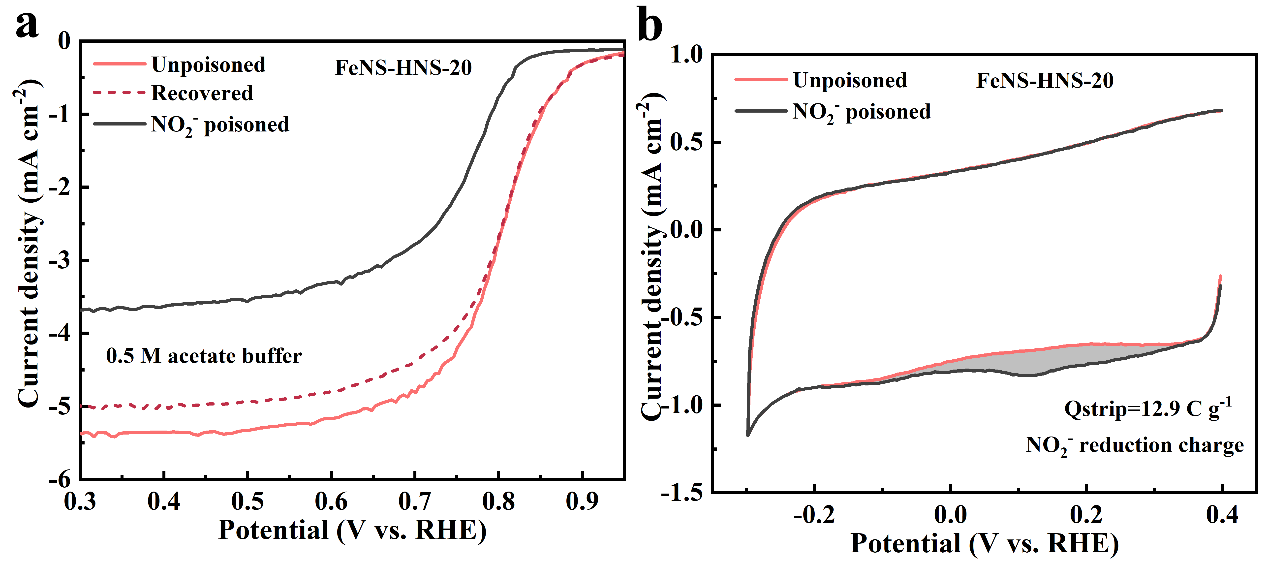


**Fig. S28** LSV and CV curves before and after nitrite adsorption for FeNS-HNS-20 in 0.5 M acetate buffer. Catalysts loading 0.27 mg cm^-2^


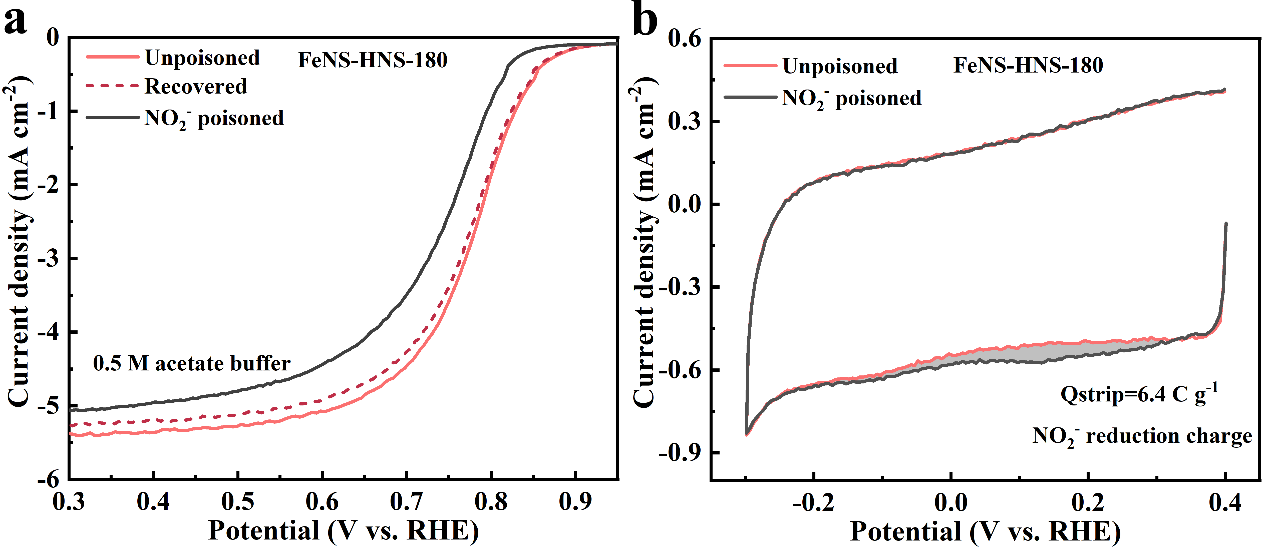


**Fig. S29** LSV and CV curves before and after nitrite adsorption for FeNS-HNS-180 in 0.5 M acetate buffer. Catalysts loading 0.27 mg cm^-2^


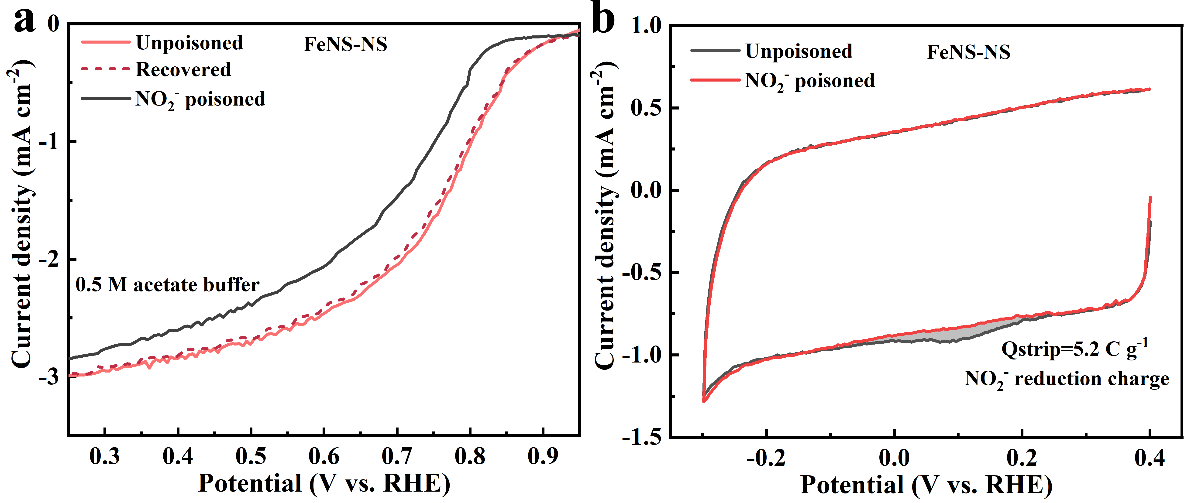


**Fig. S30** LSV and CV curves before and after nitrite adsorption for FeNS-NS in 0.5 M acetate buffer. Catalysts loading 0.27 mg cm^-2^


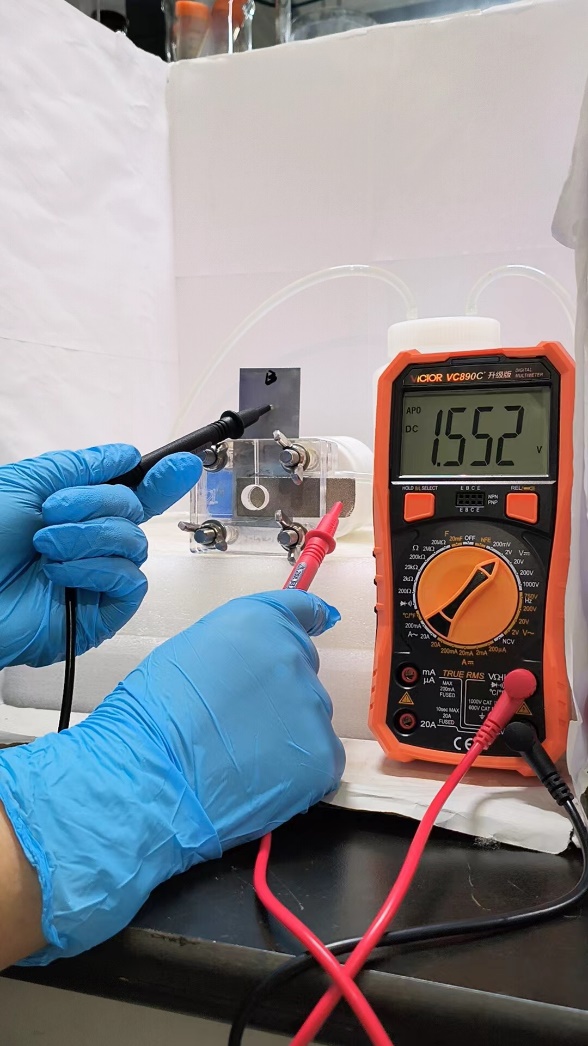


**Fig. S31** Open circuit voltage tests of rechargeable ZABs assemble with FeNS-HNS-20 as air cathodes


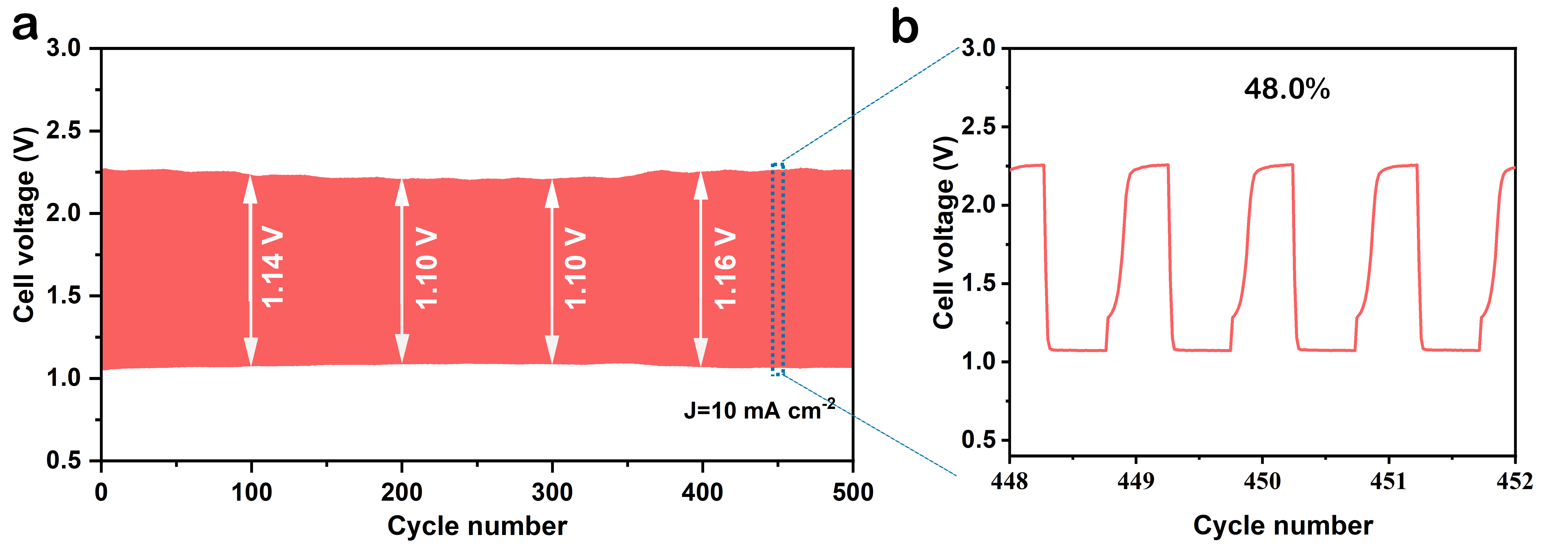


**Fig. S32 (a)** Galvanostatic cycling curves at a current density of 10 mA cm^-2^ of ZABs assembled with FeNS-HNS-20 electrocatalysts. (**b**) The zoomed-in view of voltage profiles


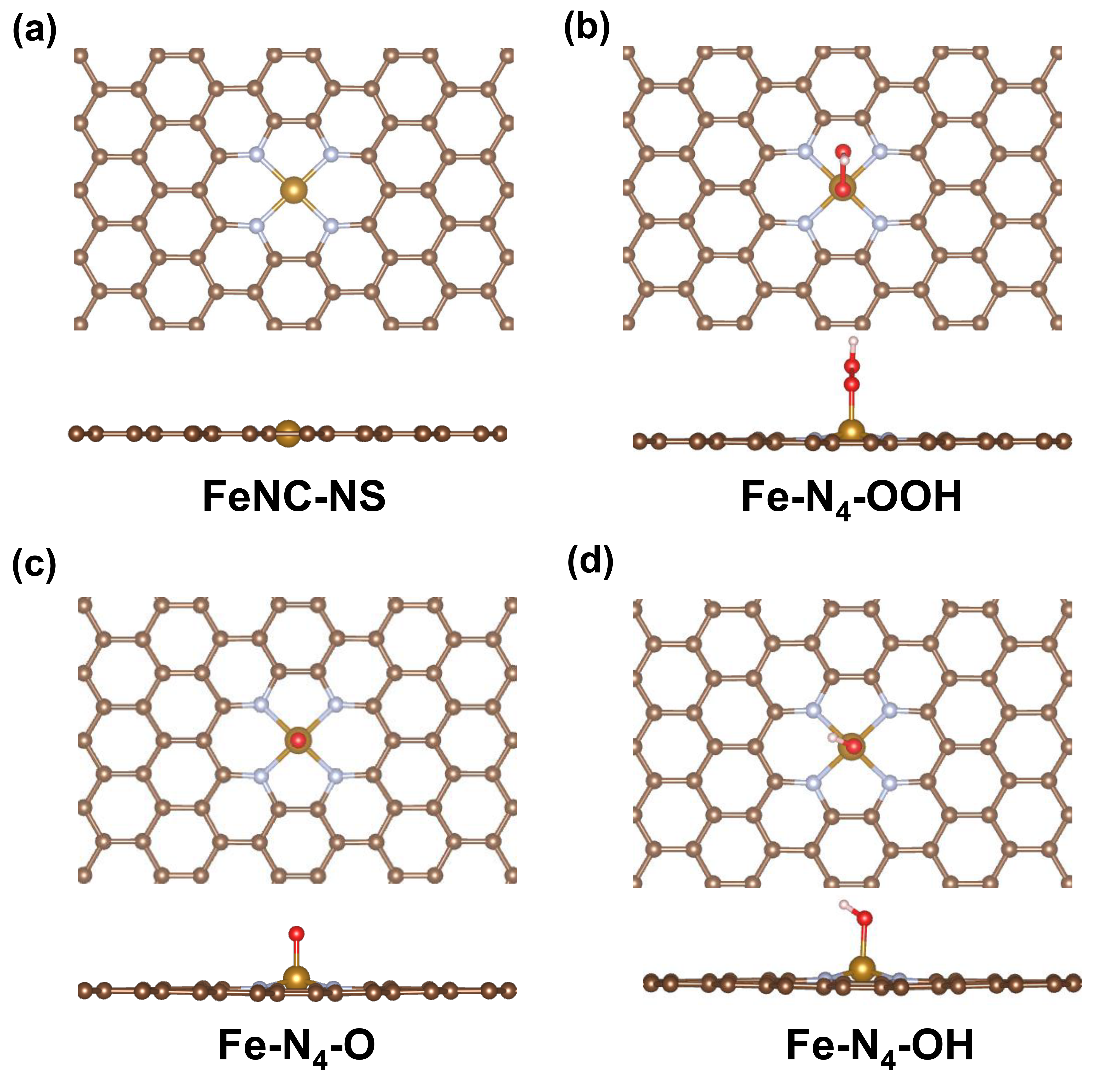


**Fig. S33** Calculation models for ORR process on FeNC nonosheets with Fe-N_4_ coordination structure (FeNC-NS)


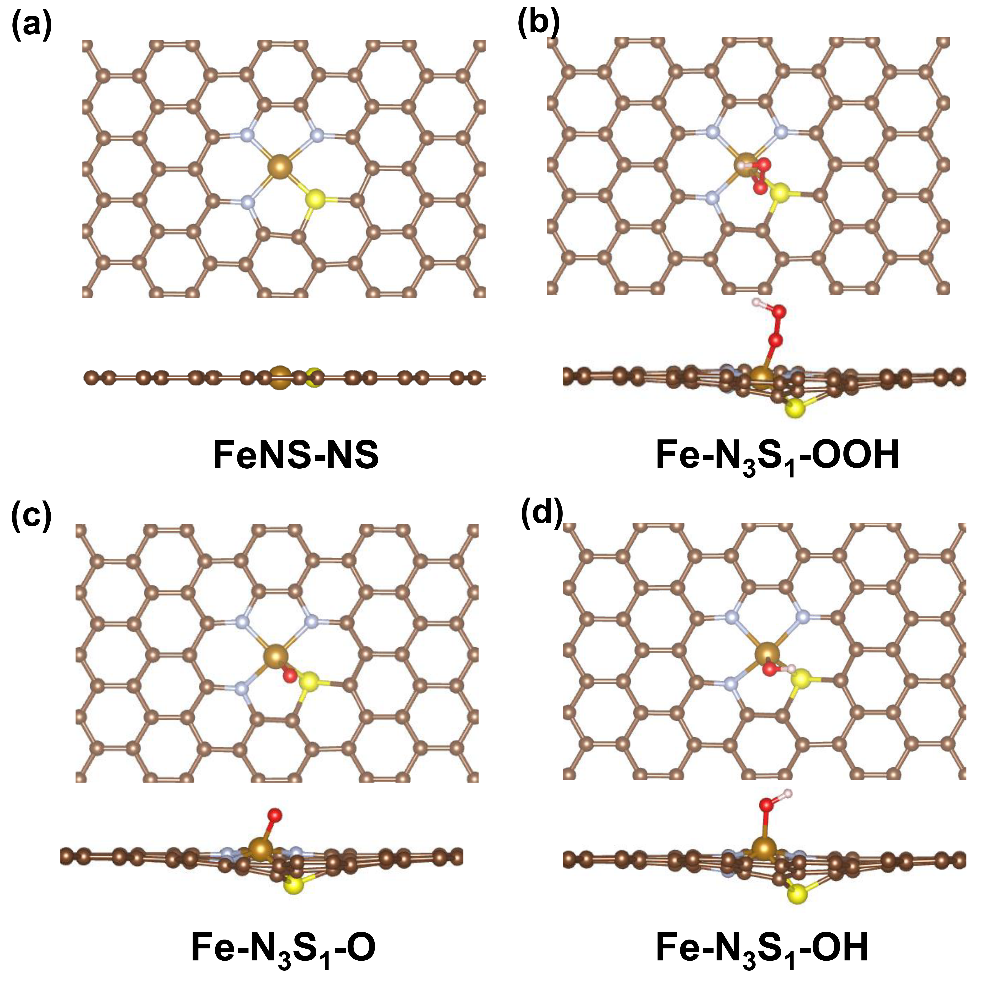


**Fig. S34** Calculation models for ORR process on FeNS nonosheets with Fe-N_3_S_1_ coordination structure (FeNS-NS)


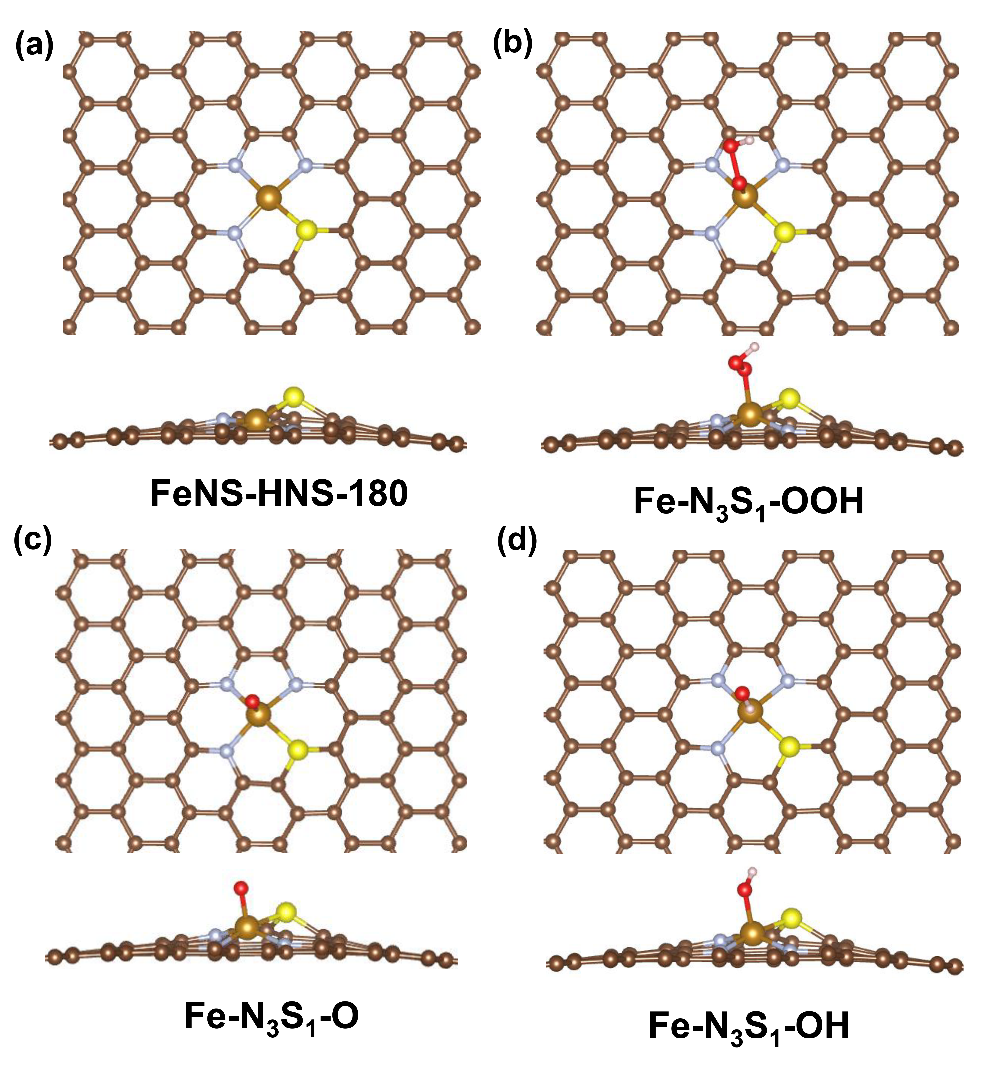


**Fig. S35** Calculation models for ORR process on Fe-N_3_S_1_ coordination structure with curvature of 0.011 (FeNS-HNS-180)


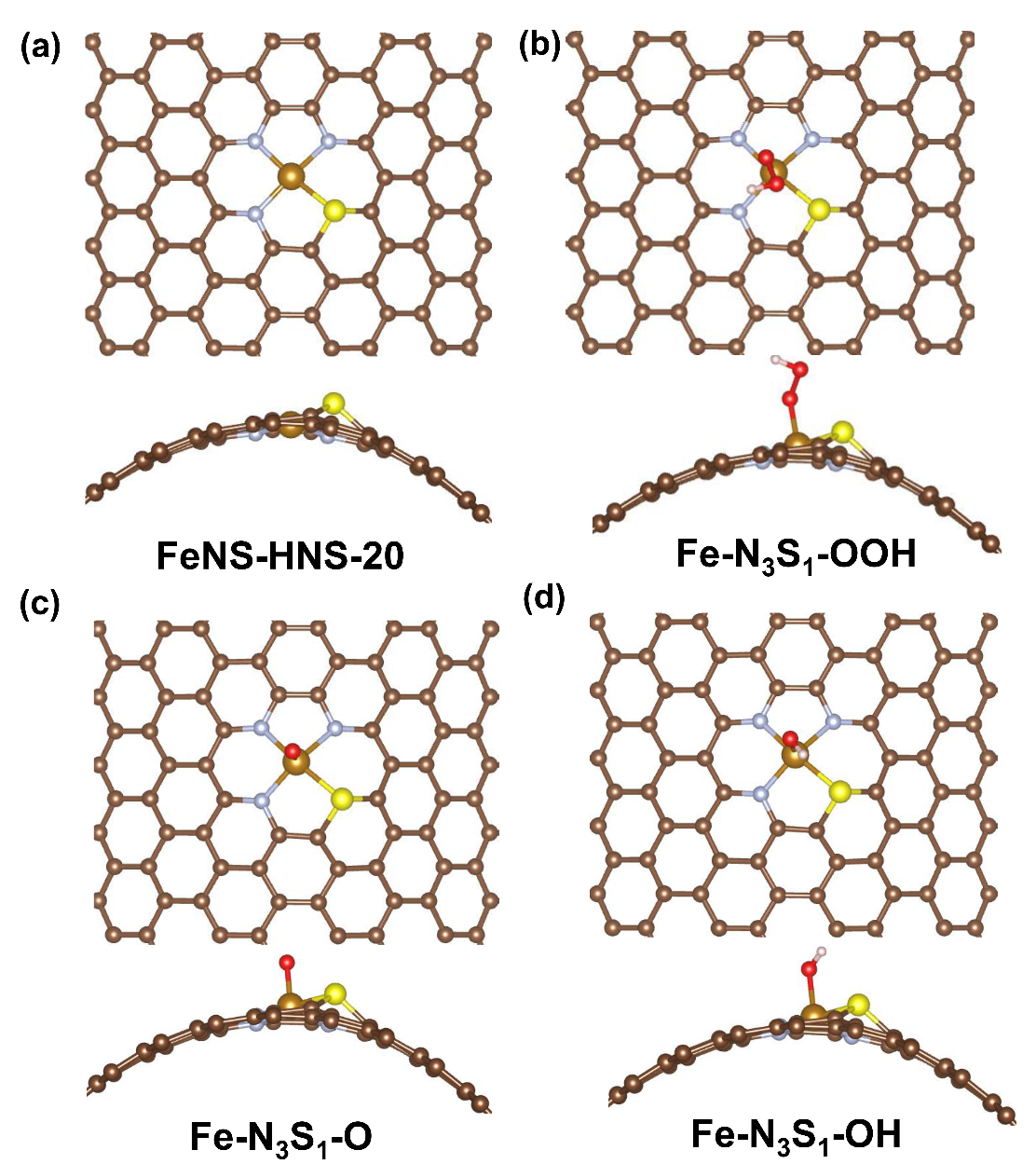


**Fig. S36** Calculation models for ORR process on Fe-N_3_S_1_ coordination structure with curvature of 0.091 (FeNS-HNS-20)


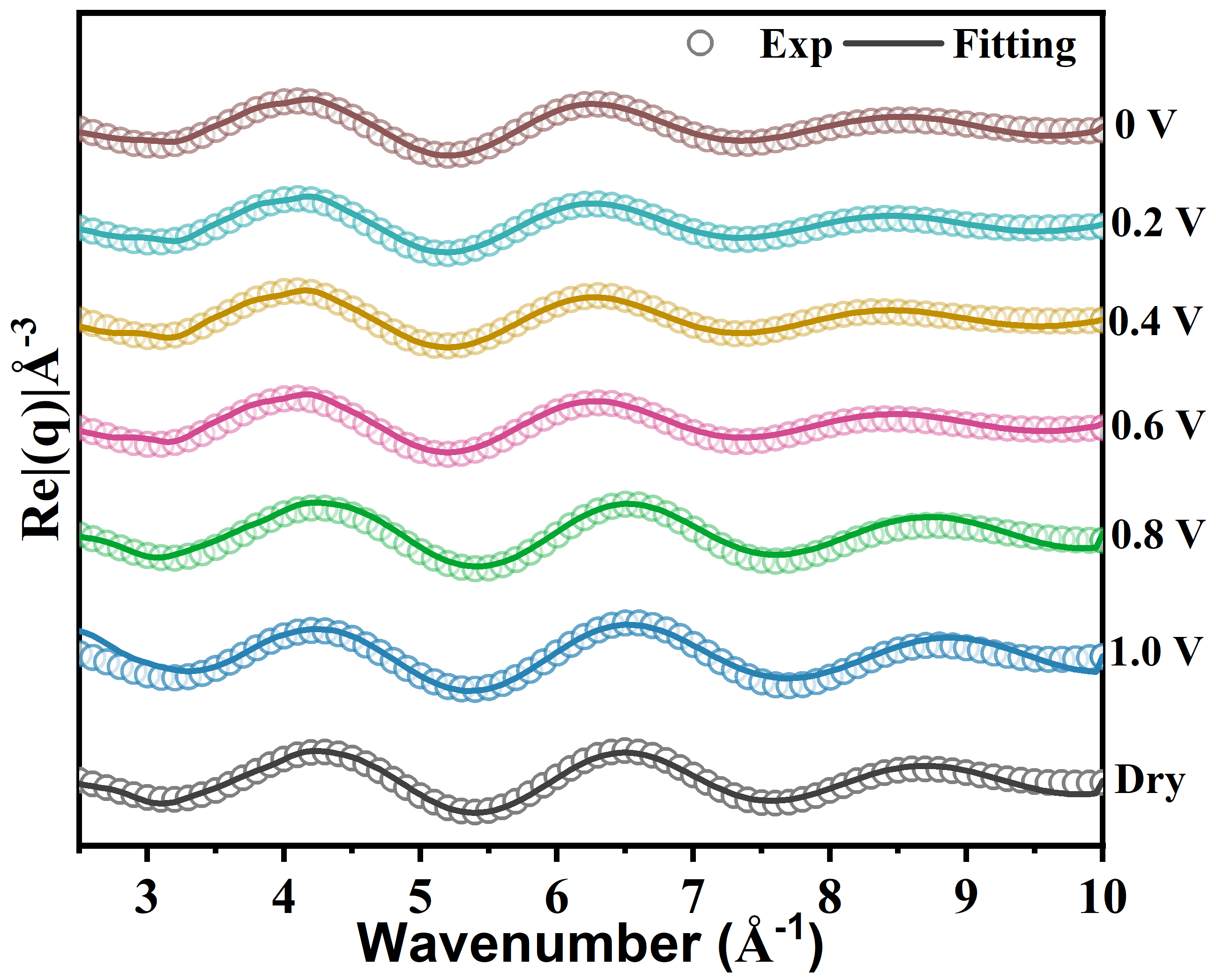


**Fig. S37** EXAFS fitting results of FeNS-HNS-20 in k space under different working potentials

**Table S1** Fitting results of FeNS-HNS-20

| Catalysts | Path | CN | R (Å) | ΔE (eV) | ΔW (eV) |
| --- | --- | --- | --- | --- | --- |
| FeNS-HNS-20 (Dry) | Fe-N | 2.9±0.3 | 1.97±0.03 | -3.2±0.5 | 0.0074±0.0007 |
|  | Fe-S | 0.9±0.2 | 2.06±0.03 | 2.5±0.5 | 0.0103±0.0005 |

**Table S2** Fe contents of as-prepared catalysts determined by ICP-OES.

| Catalysts | Fe contents (wt%) |
| --- | --- |
| FeNS-HNS-20 | 0.2964 |
| FeNS-HNS-180 | 0.3042 |
| FeNS-NS | 0.3301 |

**Table S3** Alkaline ORR activity of recently reported catalysts

| Catalysts | E_1/2_ (V vs. RHE) | E_onset_ (V vs. RHE) | Catalyst loadings (mg cm^-2^) | References |
| --- | --- | --- | --- | --- |
| FeNS-HNS-20 | 0.924 | 1.05 | 0.51 | This work |
| T-Fe SAC | 0.91 | 1.025 | 0.20 | *Angew. Chem. Int. Ed.* **2024**, *63***,** e202319370 [S10] |
| Fe/Meso-NC-1000 | 0.885 | 0.97 | 0.34 | *Adv. Mater.* **2022**, *34*, 2107291 [S11] |
| Fe-SA/PNC | 0.921 | 1.045 | 0.38 | *Angew. Chem. Int. Ed.* **2023**, *62***,** e202307504 [S12] |
| Fe-SA-NSFC | 0.91 | 1.01 | 0.50 | Nat. Commun. **2020**, *11*, 5892 [S13] |
| FePc-{PW12}@NTs | 0.90 | 1.00 | _ | *Angew. Chem. Int. Ed.* **2023**, *62***,** e202309545 [S14] |
| Fe_2_DAC | 0.898 | 1.00 | 0.57 | *Angew. Chem. Int. Ed.* **2023**, *62***,** e202304412 [S15] |
| Fe_1_Se_1_-NC | 0.88 | 1.00 | 0.20 | *Appl. Catal. B- Environ.* **2022***, 308, 121206* [S16] |
| Fe-SA/N-HCS | 0.91 | 1.02 | 0.26 | *Angew. Chem. Int. Ed.* **2023**, *62***,** e202309784 [S17] |
| FeSAs-Fe_2_P NPs/NPCFs-2.5 | 0.91 | 1.03 | 0.51 | *Adv. Mater.* **2022**, *34*, 2203621 [S18] |
| Fe-N-GDY | 0.89 | 1.05 | 0.40 | *Angew. Chem. Int. Ed.* **2022**, *61***,** e202208238 [S19] |
| FeN_3_OS | 0.874 | 1.01 | 0.40 | *Angew. Chem. Int. Ed.* **2021**, *60***,** 25296-25301 [S20] |
| Fe,P-DAS@MPC | 0.92 | 1.02 | 0.26 | *Adv. Energy Mater.* **2022**, *13*, 2203611 [S21] |
| D-Fe SAC | 0.91 | 1.015 | 0.30 | *Angew. Chem. Int. Ed.* **2021**, *60***,** 22722-22728 [S22] |
| OAC | 0.854 | 0.98 | 0.30 | *Appl. Catal. B- Environ.* **2022***, 305, 121058* [S23] |

**Table S4** Fitting results of strained FeN_3_S_1_ sites (FeNS-HNS-20) at different working potentials

| Potential  (V vs. RHE) | Path | CN | R (Å) | ΔE (eV) | ΔW (eV) |
| --- | --- | --- | --- | --- | --- |
| 1.0 V | Fe-N | 3.0 (2) | 1.97 (2) | -3.6 (6) | 0.0077 (6) |
|  | Fe-S | 1.0 (2) | 2.06 (2) | 0.6 (4) | 0.0106 (6) |
| 0.8 V | Fe-N | 2.9 (2) | 1.96 (3) | -2.7 (6) | 0.0082 (6) |
|  | Fe-S | 0.8 (3) | 2.07 (4) | 0.1 (5) | 0.0113 (6) |
| 0.6 V | Fe-N | 2.8 (2) | 1.95 (3) | -2.2 (7) | 0.0088 (6) |
|  | Fe-S | 0.7 (2) | 2.09 (2) | 0.4 (6) | 0.0116 (7) |
|  | Fe-O | 0.3 (1) | 1.96 (4) | 3.5 (5) | 0.0085 (5) |
| 0.4 V | Fe-N | 2.7 (2) | 1.94 (4) | -1.7 (6) | 0.0075 (7) |
|  | Fe-S | 0.4 (3) | 2.10 (3) | -0.4 (6) | 0.0110 (6) |
|  | Fe-O | 0.5 (2) | 1.96 (4) | 3.9 (4) | 0.0091 (5) |
| 0.2 V | Fe-N | 2.7 (3) | 1.95 (2) | -2.8 (5) | 0.0083 (6) |
|  | Fe-S | 0.2 (3) | 2.10 (3) | 1.5 (6) | 0.0120 (7) |
|  | Fe-O | 0.5 (3) | 1.96 (3) | 2.2 (5) | 0.0087 (6) |
| 0 V | Fe-N | 2.8 (2) | 1.94 (3) | -4.2 (5) | 0.0093 (6) |
|  | Fe-S | 0.2 (3) | 2.10 (4) | -1.3 (5) | 0.0108 (7) |
|  | Fe-O | 0.6 (3) | 1.95 (2) | 1.9 (6) | 0.0082 (4) |

The number in brackets represents deviation of obtained results. For example, 3.0 (2) represents the coordination number (CN) is 3.0 ± 0.2 and 1.97 (2) means the R distance is 1.97 ± 0.02.

**Supplementary References**

1. D. Malko, A. Kucernak, T. Lopes, *In situ* electrochemical quantification of active sites in Fe-N/C non-precious metal catalysts. Nat. Commun. **7**, 13285 (2016). <https://doi.org/10.1038/ncomms13285>
2. G. Kresse, J. Furthmüller, Efficiency of ab-initio total energy calculations for metals and semiconductors using a plane-wave basis set. Comput. Mater. Sci. **6**(1), 15–50 (1996). <https://doi.org/10.1016/0927-0256(96)00008-0>
3. G. Kresse, J. Furthmüller, Efficient iterative schemes for *ab initio* total-energy calculations using a plane-wave basis set. Phys. Rev. B Condens. Matter **54**(16), 11169–11186 (1996). <https://doi.org/10.1103/physrevb.54.11169>
4. J.P. Perdew, K. Burke, M. Ernzerhof, Generalized gradient approximation made simple. Phys. Rev. Lett. **77**(18), 3865–3868 (1996). <https://doi.org/10.1103/physrevlett.77.3865>
5. G. Kresse, D. Joubert, From ultrasoft pseudopotentials to the projector augmented-wave method. Phys. Rev. B **59**(3), 1758–1775 (1999). <https://doi.org/10.1103/physrevb.59.1758>
6. S. Grimme, Semiempirical GGA-type density functional constructed with a long-range dispersion correction. J. Comput. Chem. **27**(15), 1787–1799 (2006). <https://doi.org/10.1002/jcc.20495>
7. H.J. Monkhorst, J.D. Pack, Special points for Brillouin-zone integrations. Phys. Rev. B **13**(12), 5188–5192 (1976). <https://doi.org/10.1103/physrevb.13.5188>
8. J.K. Nørskov, J. Rossmeisl, A. Logadottir, L. Lindqvist, J.R. Kitchin et al., Origin of the overpotential for oxygen reduction at a fuel-cell cathode. J. Phys. Chem. B **108**(46), 17886–17892 (2004). <https://doi.org/10.1021/jp047349j>
9. J. Rossmeisl, A. Logadottir, J.K. Nørskov, Electrolysis of water on (oxidized) metal surfaces. Chem. Phys. **319**(1–3), 178–184 (2005). <https://doi.org/10.1016/j.chemphys.2005.05.038>
10. Y. Zhu, Y. Jiang, H. Li, D. Zhang, L. Tao et al., Tip-like Fe−N4 sites induced surface microenvironments regulation boosts the oxygen reduction reaction. Angew. Chem. Int. Ed. **63**(11), e202319370 (2024). <https://doi.org/10.1002/anie.202319370>
11. S.-N. Zhao, J.-K. Li, R. Wang, J. Cai, S.-Q. Zang, Electronically and geometrically modified single-atom Fe sites by adjacent Fe nanoparticles for enhanced oxygen reduction. Adv. Mater. **34**(5), 2107291 (2022). <https://doi.org/10.1002/adma.202107291>
12. W. Xue, Q. Zhou, X. Cui, J. Zhang, S. Zuo et al., Atomically dispersed FeN_2_P_2_ motif with high activity and stability for oxygen reduction reaction over the entire pH range. Angew. Chem. Int. Ed. **62**(41), e202307504 (2023). <https://doi.org/10.1002/anie.202307504>
13. Y. Zhou, X. Tao, G. Chen, R. Lu, D. Wang et al., Multilayer stabilization for fabricating high-loading single-atom catalysts. Nat. Commun. **11**(1), 5892 (2020). <https://doi.org/10.1038/s41467-020-19599-8>
14. S. Zhu, L. Ding, X. Zhang, K. Wang, X. Wang et al., Biaxially-strained phthalocyanine at Polyoxometalate@Carbon nanotube heterostructure boosts oxygen reduction catalysis. Angew. Chem. Int. Ed. **62**(42), e202309545 (2023). <https://doi.org/10.1002/anie.202309545>
15. W.-D. Zhang, L. Zhou, Y.-X. Shi, Y. Liu, H. Xu et al., Dual-atom catalysts derived from a preorganized covalent organic framework for enhanced electrochemical oxygen reduction. Angew. Chem. Int. Ed. **62**(27), e202304412 (2023). <https://doi.org/10.1002/anie.202304412>
16. Z. Chen, X. Su, J. Ding, N. Yang, W. Zuo et al., Boosting oxygen reduction reaction with Fe and Se dual-atom sites supported by nitrogen-doped porous carbon. Appl. Catal. B Environ. **308**, 121206 (2022). <https://doi.org/10.1016/j.apcatb.2022.121206>
17. L. Zong, K. Fan, L. Cui, F. Lu, P. Liu et al., Constructing Fe-N4 sites through anion exchange-mediated transformation of Fe coordination environments in hierarchical carbon support for efficient oxygen reduction. Angew. Chem. Int. Ed. **62**(38), e202309784 (2023). <https://doi.org/10.1002/anie.202309784>
18. Y. Pan, X. Ma, M. Wang, X. Yang, S. Liu et al., Construction of N, P Co-doped carbon frames anchored with Fe single atoms and Fe_2_P nanoparticles as a robust coupling catalyst for electrocatalytic oxygen reduction. Adv. Mater. **34**(29), 2203621 (2022). <https://doi.org/10.1002/adma.202203621>
19. M. Li, Q. Lv, W. Si, Z. Hou, C. Huang, Sp-hybridized nitrogen as new anchoring sites of iron single atoms to boost the oxygen reduction reaction. Angew. Chem. Int. Ed. **61**(38), e202208238 (2022). <https://doi.org/10.1002/anie.202208238>
20. L. Yu, Y. Li, Y. Ruan, Dynamic control of sacrificial bond transformation in the Fe−N−C single-atom catalyst for molecular oxygen reduction. Angew. Chem. Int. Ed. **60**(48), 25296–25301 (2021). <https://doi.org/10.1002/anie.202111761>
21. L. Zong, K. Fan, P. Li, F. Lu, B. Li et al., Promoting oxygen reduction reaction on atomically dispersed Fe sites *via* establishing hydrogen bonding with the neighboring P atoms. Adv. Energy Mater. **13**(5), 2203611 (2023). <https://doi.org/10.1002/aenm.202203611>
22. J. Yang, Z. Wang, C.-X. Huang, Y. Zhang, Q. Zhang et al., Compressive strain modulation of single iron sites on helical carbon support boosts electrocatalytic oxygen reduction. Angew. Chem. Int. Ed. **60**(42), 22722–22728 (2021). <https://doi.org/10.1002/anie.202109058>
23. L. Deng, L. Qiu, R. Hu, L. Yao, Z. Zheng et al., Restricted diffusion preparation of fully-exposed Fe single-atom catalyst on carbon nanospheres for efficient oxygen reduction reaction. Appl. Catal. B Environ. **305**, 121058 (2022). <https://doi.org/10.1016/j.apcatb.2021.121058>
